# Compensatory variability in network parameters enhances memory performance in the *Drosophila* mushroom body

**DOI:** 10.1073/pnas.2102158118

**Published:** 2021-11-29

**Authors:** Nada Y. Abdelrahman, Eleni Vasilaki, Andrew C. Lin

**Affiliations:** ^a^School of Biosciences, University of Sheffield, Sheffield S10 2TN, United Kingdom;; ^b^Department of Computer Science, University of Sheffield, Sheffield S1 4DP, United Kingdom;; ^c^Neuroscience Institute, University of Sheffield, Sheffield S10 2TN, United Kingdom

**Keywords:** *Drosophila*, mushroom body, homeostatic plasticity, associative memory

## Abstract

Neural circuits use homeostatic compensation to achieve consistent behavior despite variability in underlying intrinsic and network parameters. However, it remains unclear how compensation regulates variability across a population of the same type of neurons within an individual and what computational benefits might result from such compensation. We address these questions in the Drosophila mushroom body, the fly’s olfactory memory center. In a computational model, we show that under sparse coding conditions, memory performance is degraded when the mushroom body’s principal neurons, Kenyon cells (KCs), vary realistically in key parameters governing their excitability. However, memory performance is rescued while maintaining realistic variability if parameters compensate for each other to equalize KC average activity. Such compensation can be achieved through both activity-dependent and activity-independent mechanisms. Finally, we show that correlations predicted by our model’s compensatory mechanisms appear in the Drosophila hemibrain connectome. These findings reveal compensatory variability in the mushroom body and describe its computational benefits for associative memory.

## Significance

How does variability between neurons affect neural circuit function? How might neurons behave similarly despite having different underlying features? We addressed these questions in neurons called Kenyon cells, which store olfactory memories in flies. Kenyon cells differ among themselves in key features that affect how active they are, and in a model of the fly’s memory circuit, adding this interneuronal variability made the model fly worse at learning the values of multiple odors. However, memory performance was rescued if compensation between the variable underlying features allowed Kenyon cells to be equally active on average, and we found the hypothesized compensatory variability in real Kenyon cells’ anatomy. This work reveals the existence and computational benefits of compensatory variability in neural networks.

Noise and variability are inevitable features of biological systems. Neural circuits achieve consistent activity patterns despite this variability using homeostatic plasticity; because neural activity is governed by multiple intrinsic and network parameters, variability in one parameter can compensate for variability in another to achieve the same circuit behavior (1–5). This phenomenon of compensatory variability has typically been addressed from the perspective of consistency of neural activity across individual animals (6, 7) or over an animal’s lifetime, in the face of circuit perturbations (8–11). However, less attention has been paid to potential benefits of maintaining consistent neuronal properties across a population of neurons within an individual circuit.

Indeed, previous work has emphasized the benefits of neuronal variability/heterogeneity rather than neuronal homogeneity (12–14). (Here, we follow ref. 5 in using “heterogeneity” to refer to qualitative differences [e.g., between cell types] and “variability” to refer to quantitative differences in parameter values.) Of course, different neuronal classes encode different information (e.g., visual vs. auditory neurons or ON vs. OFF cells). Yet, even in populations that ostensibly encode the same kind of stimulus, like olfactory mitral cells, variability of neuronal excitability can increase the information content of their population activity (15–17). In addition, variability in neuronal timescales can improve learning in neural networks (18, 19). In what contexts and in what senses might the opposite be true (i.e., when does neuronal similarity provide computational benefits over neuronal variability)? Additionally, what mechanisms could enforce neuronal similarity in the face of interneuronal variability?

Here, we address these questions using olfactory associative memory in the mushroom body of the fruit fly *Drosophila*. Flies learn to associate specific odors with salient events (e.g., food or danger). These olfactory associative memories are stored in the principal neurons of the mushroom body, called Kenyon cells (KCs), as modifications in KCs’ output synapses (20–22) (reviewed in ref. 23). Because learning occurs at the single output layer, the nature of the odor representation in the KC population is crucial to the fly’s ability to learn to form distinct associative memories for different odors. In particular, the fact that KCs respond sparsely to incoming odors (≈ 10% per odor) (24) allows different odors to activate unique, nonoverlapping subsets of KCs and thereby enhances flies’ learned discrimination of similar odors (25).

A potential problem for this sparse coding arises from variability between KCs. KCs receive inputs from second-order olfactory neurons called projection neurons (PNs), with an average of approximately six PN inputs per KC, and typically require simultaneous activation of multiple input channels in order to spike (26), thanks to high spiking thresholds and feedback inhibition (25, 27). However, there is substantial variation across KCs in the key parameters controlling their activity, such as the number of PN inputs per KC (28), the strength of PN–KC synapses, and KC spiking thresholds (27). Intuitively, such variation could lead to a situation where some KCs with low spiking thresholds and many or strong excitatory inputs fire indiscriminately to many different odors, while other KCs with high spiking thresholds and few or weak excitatory inputs never fire; KCs at both extremes are effectively useless for learning to classify odors, even if overall only 10% of KCs respond to each odor. However, it remains unclear whether biologically realistic inter-KC variability would affect the mushroom body’s memory performance and what potential strategies might counter the effects of inter-KC variability.

Here, we show in a rate-coding model of the mushroom body that introducing experimentally derived inter-KC variability into the model substantially impairs its memory performance. This impairment arises from increased variability in average activity among KCs, which means fewer KCs have sparse-enough activity to be specific to rewarded vs. punished odors. However, memory performance can be rescued by compensating away variability in KC activity while preserving the experimentally observed variation in the underlying parameters. This can occur through activity-dependent homeostatic plasticity or direct correlations between key parameters like number vs. strength of inputs. Finally, we analyze the hemibrain connectome to show that, indeed, the number of PN inputs per KC is inversely correlated with the strength of each input, while the strength of inhibitory inputs is correlated with the total strength of excitatory inputs. Thus, we show both the existence and computational benefit of compensatory variability in mushroom body network parameters.

## Results

### Realistic Inter-KC Variability Impairs Memory Performance under Sparse Coding

**Fig. 1.**
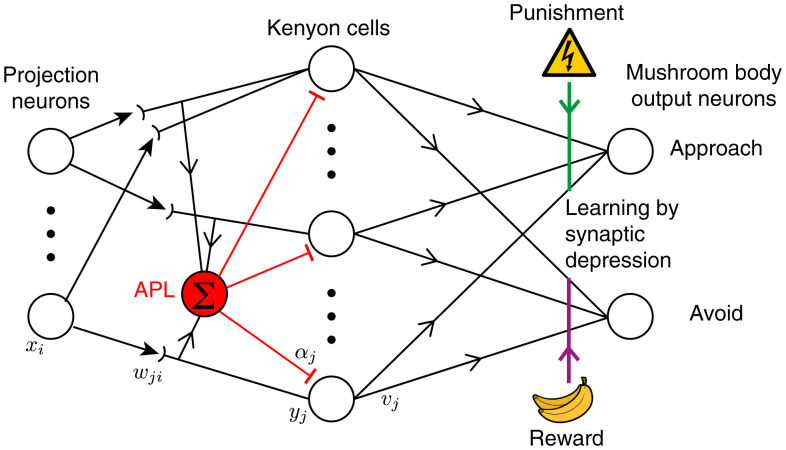
Schematic for the mushroom body network model. PNs in the input layer relay the odor responses, *x_i_*, downstream to the KCs (*y_j_*). KCs connect randomly to the PNs with synaptic weights *w_ji_* and receive global inhibition from the APL neuron with weight *α_j_*. Learning occurs when DANs carrying punishment (reward) signals from the environment depress the synapses (*v_j_*) between the active KCs and the MBONs that lead to approach (avoidance) behavior.

To study how variability between KCs might affect the fly’s olfactory memory performance, we modeled the mushroom body as a rate-coding neural network (Fig. 1). To simulate the input activity from PNs, we modeled their activity as a saturating nonlinear function of activity of the first-order olfactory receptor neurons (ORNs) (*SI Appendix* ) (29). We applied this function to the recorded odor responses of 24 different olfactory receptors (30) to yield simulated PN activity, as in previous computational studies of fly olfaction (31–34). To simulate variability in PN activity across different encounters with the same odor, we created several “trials” of each odor and added Gaussian noise to PN activity, following the coefficients of variation reported in ref. 35. To increase the number of stimuli beyond the 110 recorded odors in ref. 30, we generated odor responses in which the activity of each PN was randomly sampled from that PN’s activity across the 110 odors used in ref. 30 (results were similar with the “real” 110 odors) (*SI Appendix*; see below).

Each of the 2,000 KCs in our model received excitatory input from a randomly selected set of *N* PNs, each with strength *w*. A KC’s response to each odor was the sum of excitatory inputs minus inhibition, minus a spiking threshold *θ*; if net excitation was below the threshold, the activity was set to zero. Inhibition came from the feedback interneuron APL (“anterior paired lateral”), which is excited by and inhibits all KCs (25). To avoid simulating the network in time, we simplified the feedback inhibition into pseudofeedforward inhibition, in which APL’s activity was the sum of all postsynaptic excitation of all KCs (without the KCs’ threshold applied); we based this simplification on the fact that KCs and APL form reciprocal synapses with each other on KC dendrites (i.e., before the KCs’ spike initiation zone), and APL activity is somewhat spatially restricted between KC axons and dendrites (36). Thresholds and inhibition were scaled so that on average 10% of KCs were active for each odor (“coding level” = 0.1).

Learning in flies occurs when KCs (responding to odor) are active at the same time as dopaminergic neurons (DANs; responding to “reward” or “punishment”); the coincident activity modifies the output synapse from KCs onto mushroom body output neurons (MBONs) that lead to behavior (e.g., approaching or avoiding an odor). Typically, the output to the “wrong” behavior is depressed; for example, pairing an odor with electric shock weakens the output synapses from KCs activated by that odor onto MBONs that lead to “approach” behavior (21, 22, 37, 38) (reviewed in ref. 23). We simulated this plasticity using a simplified architecture with only two MBONs: “approach” and “avoid.” The input odors were randomly divided; half were paired with punishment, and half were paired with reward. During training, KCs activated by rewarded odors weakened their synapses onto the avoid MBON, while KCs activated by punished odors weakened their synapses onto the approach MBON (depression by exponential decay; see *SI Appendix*). The fly’s behavior then depended probabilistically (via a softmax function; see *SI Appendix*) on whether the avoid or approach MBON’s activity was greater, and the model’s accuracy in learning was scored as the fraction of correct decisions for unseen noisy variants of the trained odors (i.e., avoiding punished odors and approaching rewarded odors).

**Fig. 2.**
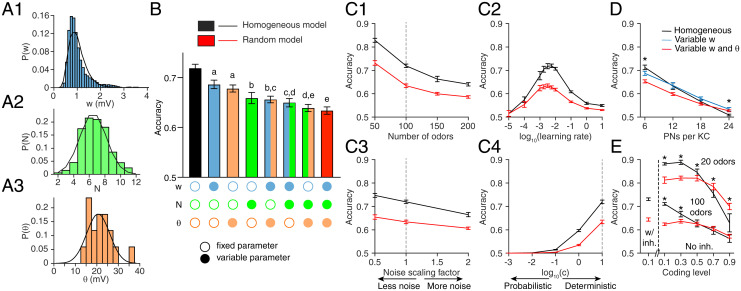
Inter-KC variability in w,N, and *θ* degrades the model fly’s memory performance. (*A*) Histograms of the experimentally measured distributions for (*A*1) w (amplitude of spontaneous excitatory postsynaptic potentials in KCs; millivolts) (data are from ref. 27), (*A2*) N (number of PN inputs per KC; measured as the number of dendritic “claws”) (data are from ref. 28), and (*A*3) *θ* (spiking threshold minus resting potential; mV) (data are from ref. 27). The overlaid black curves show log-normal (w) and Gaussian (N, *θ*) fits to the data. (*B*) The model fly’s memory performance (given 100 input odors), varying the parameters step by step. Fixed and variable parameters are shown by empty and filled circles, respectively. The homogeneous model (all parameters fixed, N=6; black) performs the best, and the random model (all parameters variable; red) performs the worst. All bars are significantly different from each other unless they share the same letter annotations (a, b, etc.). *P* < 0.05 by Wilcoxon signed rank test (for matched models with the same PN–KC connectivity) or Mann–Whitney test (for unmatched models with different PN–KC connectivity; i.e., fixed vs. variable N), with Holm–Bonferroni correction for multiple comparisons (full statistics are in Dataset S1). *n* = 30 model instances with different random PN–KC connectivity. (*C*) The performance trend is consistent over a range of different conditions: (*C*1) the number of input odors; (*C*2) the learning rate used to update KC–MBON weights; (*C*3) the amount of noise in PN activity (half, the same, or double the noise measured in ref. 35); and (*C*4) the indeterminacy in the decision making, quantified by log(c), where c is the constant in the softmax function (*SI Appendix*, Eq. 21). The vertical dotted lines indicate the conditions used in *B* (each condition used the best learning rate). (*D*) As KCs receive more inputs (thus, more similar inputs), inter-KC variability becomes helpful, not harmful, to memory performance, especially when all KCs receive the same inputs (N=24). Blue, KCs vary in excitatory weights (w); red, KCs vary in both w and thresholds (*θ*). Data for N=6 are equivalent to *B*. n=30. (*E*) Inter-KC variability improves performance in dense coding regimes (coding levels 0.7 to 0.9) at classifying 100 odors (a hard task) or 20 odors (easy task). Left of the dashed line is equivalent to *B* for comparison. Right of the dashed line is increasing coding levels, in each case without inhibition (because inhibition is constrained to decrease coding level by half, which is impossible if coding level >0.5). *n* = 50. Error bars show 95% CIs. *P < 0.05, Wilcoxon signed rank test (*D*) or Mann–Whitney test (*E*) with Holm–Bonferroni correction for multiple comparisons.

To test the effect of realistic inter-KC variability on this model, we introduced variability step by step. We first tested the performance of the model holding constant across all KCs the three parameters *N* (number of PN inputs per KC), *w* (strength of each PN–KC connection), and *θ* (KC spiking threshold). Then, we added inter-KC variability step by step: first varying only one of three parameters, then varying two of three, and finally varying all three parameters (thus, eight possible models). Inter-KC variability in *N*, *w*, and *θ* followed experimentally measured distributions (Fig. 2 *A1–A3*) (27, 28). Increasing inter-KC variability systematically degraded the model’s performance when tested on 100 input odors; the more variable parameters there were, the worse the performance (Fig. 2*B*). This performance trend was the same when these eight models were trained and tested on the real input odors responses from ref. 30 (*SI Appendix*, Fig. S1*A*).

**Fig. 3.**
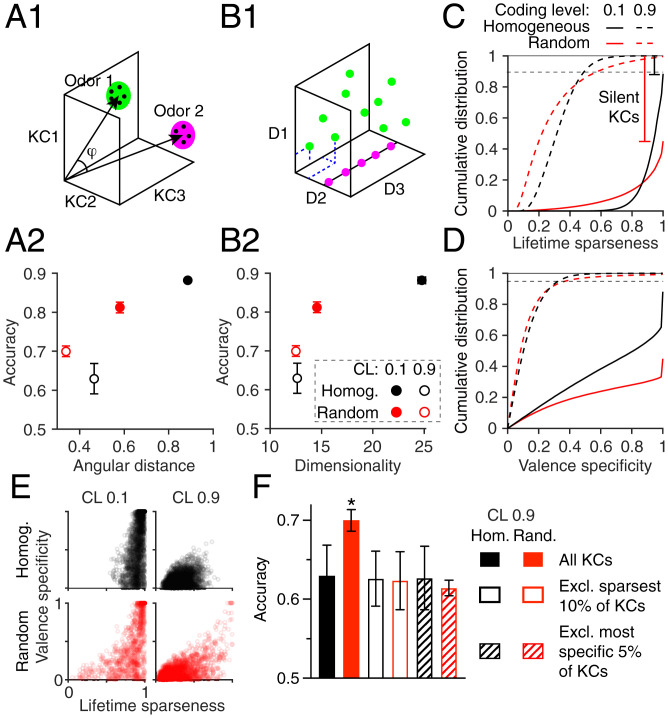
Performance depends on KC lifetime sparseness. (*A*1 and *B*1) Diagrams of angular distance between odors (i.e., between centroids of clusters of noisy trials; *A*1) and dimensionality of a system with three variables (*B*1). The system with its states scattered throughout three-dimensional space (green) has dimensionality 3, while the system with all states on a single line (magenta) has dimensionality 1. (*A*2 and *B*2) The homogeneous model has higher angular distance and dimensionality than the random model (*P* < 0.05, Mann–Whitney test), matching the performance difference when coding level is 0.1 but the opposite trend to performance when coding level is 0.9. CL, coding level; Homog., homogeneous. (*C* and *D*) cdf of the lifetime sparseness (*C*) or valence specificity (*D*) of KCs in the homogeneous (black) and random (red) models across 50 model instantiations. The gap between 1.0 and the top of the cdf represents silent KCs (lifetime sparseness and specificity undefined). At coding level 0.1, the random model has many more silent KCs, nonsparse KCs, and nonspecific KCs than the homogeneous model, but at coding level 0.9, the random model has more KCs with high lifetime sparseness and more KCs with high valence specificity. (*E*) High lifetime sparseness enables high valence specificity, although many sparse KCs have low valence specificity because of random valence assignments (data here are from single model instances). (*F*) Removing the sparsest or most valence-specific KCs (corresponding to the dashed horizontal lines in *C* and *D*) removes the performance advantage of the random model under dense coding. Hom., homogeneous; Rand., random. n=50 network instantiations. Error bars are 95% CIs (horizontal error bars in *A*2 and *B*2 are smaller than the symbols). These results are from the 20-odor task in Fig. 2*E*; *SI Appendix*, Fig. S2 shows results of the 100-odor task. *P < 0.05, Mann–Whitney test (Dataset S1).

To test whether this effect is robust to different learning and testing conditions, we tested the two extreme cases while varying the numbers of input odors to be classified, the amount of noise in PN activity, the learning rate at the KC–MBON synapse (the two models might have different optimal learning rates) (*η* in *SI Appendix*, Eq. 20), or the indeterminacy of the fly’s decision making (*c* in *SI Appendix*, Eq. 21). In every case, the model with all parameters fixed (which we call the “homogeneous” model) consistently outperformed the model with all parameters variable (which we call the “random” model) (Fig. 2 *C1–C4*). These results indicate that biologically realistic variability in KC network parameters impairs the network’s ability to classify odors as rewarded vs. punished.

Our conclusion contrasts with earlier results that interneuronal variability between mitral cells increases information content (15–17) (i.e., that variability is helpful, not harmful). This apparent contradiction can be resolved by noting two differences between our approaches. First, the mitral cell studies provided the same input to every neuron, whereas here, every KC receives different inputs thanks to random PN–KC connectivity. Indeed, when we forced every KC to receive input from the same PNs (*N* = 24; i.e., every KC receives input from every PN) (Fig. 2*D*), variability between KCs in input weights actually improved performance compared with the homogeneous model (although both models unsurprisingly performed much worse compared with the more realistic *N* = 6). In other words, when all KCs receive the same input, only inter-KC variability allows them to have different odor response profiles from each other (39), which is required for distinct olfactory memories to be formed at KC output synapses.

Second, unlike in our model, the mitral cell studies did not enforce sparse coding where only a small fraction of cells should respond at any given time. Indeed, under dense coding (coding level = 0.9), while all models unsurprisingly performed worse than under sparse coding (coding level = 0.1), the random model outperformed the homogeneous model. While this difference was only marginal when discriminating 100 odors (possibly due to a floor effect), it was more apparent on an easier task where the network learned to classify 20 odors instead of 100 (Fig. 2*E*). Thus, while sparse coding and diverse PN inputs for each KC greatly improve learned odor classification, these features require homogeneous KCs to fully exploit their advantages, thus making inter-KC variability harmful rather than helpful under sparse coding.

### Performance Depends on KC Lifetime Sparseness

We next asked what features of KC population odor representations might account for the worse performance of the random model compared with the homogeneous model under sparse coding but the reverse under dense coding. Learning KC–MBON weights to correctly classify rewarded vs. punished odors is equivalent to finding a hyperplane (in 2,000-dimensional space) to separate KC responses to rewarded odors from those to punished odors. Finding a separating hyperplane might be easier if 1) odors are far apart from each other in KC coding space (measured by angular distance, a scale-insensitive distance metric [Fig. 3*A*1] used in, e.g., ref. 27) or 2) odor responses occupy more independent dimensions (measured by a metric for dimensionality developed by ref. 39) (Fig. 3*B*1). Indeed, under sparse coding (coding level = 0.1), the random model had smaller angular distances and lower dimensionality than the homogeneous model (Fig. 3 *A* and *B* and *SI Appendix*, Fig. S2). However, surprisingly, the same was true at coding level = 0.9, even though in this condition, the random model outperformed the homogeneous model (Fig. 2*E*), suggesting that separation and dimensionality of KC odor responses alone do not explain inter-KC variability’s effect on performance, at least with the learning rule used here (i.e., depression of KC outputs to wrong actions by exponential decay).

Instead, we hypothesized that inter-KC variability impairs performance under sparse coding because it makes some KCs indiscriminately active but leaves others completely silent, meaning fewer KCs provide useful odor identity information. Sparse coding requires sparseness in two dimensions: population sparseness (each stimulus activates few neurons) and lifetime sparseness (each neuron responds to few stimuli) (40). While our models enforced population sparseness (coding level = 0.1), they did not enforce any particular lifetime sparseness. In an extreme case, a model could have very consistent population sparseness with a coding level of 0.1 for all odors simply by having the same 10% of cells responding equally to every odor and the other 90% being completely silent. In this case, no cells would provide any useful information about odor identity. We asked whether a less extreme version of this problem could explain the relative performance of our models.

To test this, we quantified the specificity of KCs both across all odors and for rewarded vs. punished odors. To quantify specificity across odors, we used lifetime sparseness, a metric that is 1 when a cell fires to one stimulus and no other stimuli vs. 0 when it fires equally to all stimuli. A cell that fires to no stimuli has an undefined sparseness (*SI Appendix*). The homogeneous model had fairly consistent lifetime sparseness values, with almost 80% of KCs having a lifetime sparseness between ∼ 0.85 and 1. In contrast, the random model had KCs with much more variable lifetime sparseness, with a long tail of KCs with low sparseness (below 0.7) and more than 50% of KCs having undefined sparseness (i.e., completely silent). (These figures are when considering 20 odors; when considering 100 odors, there are fewer silent KCs, but the overall pattern is the same [*SI Appendix*, Fig. S2].) The contrasting distributions of lifetime sparseness can be seen in the cumulative distribution functions (cdfs) of lifetime sparseness in Fig. 3*C* and *SI Appendix*, Fig. S2*F* in how the steep curve of the homogeneous model and the shallow curve of the random model cross each other. This result can also be seen in the larger SD of lifetime sparseness across KCs in the random model (*SI Appendix*, Fig. S2 *D* and *E*). The silent KCs can be seen as the fraction of missing KCs needed for the cdf curves to reach 1; the random model has many more silent KCs than the homogeneous model.

To quantify KCs’ specificity for rewarded vs. punished odors, we defined “valence specificity” for each KC as the absolute value of the difference between total activity for all rewarded vs. all punished odors, divided by total activity for all odors. Again, under sparse coding, the homogeneous model had more KCs with high valence specificity than the random model (Fig. 3*D*). Given random valence assignments, high lifetime sparseness does not guarantee high valence specificity but does make it more probable (the two measures are correlated [Fig. 3*E*]) for the same reason that flipping a coin 5 times is more likely to give all heads than flipping a coin 50 times; a KC active for only a few odors is more likely to be active only for rewarded (or punished) odors, compared with a KC active for many odors.

Under dense coding, KCs also have more variable lifetime sparseness in the random model (dashed lines in Fig. 3*C* and *SI Appendix*, Fig. S2). However, here, the inter-KC variability is helpful rather than harmful; whereas KCs in the homogeneous model have uniformly low lifetime sparseness (and thus, are uniformly useless for odor discrimination), in the random model, the inter-KC variability allows a small minority of KCs to have relatively high lifetime sparseness and valence specificity (although still worse than under sparse coding) (Fig. 3 *C–E*). To test whether this minority of relatively specific KCs explains the better performance of the random model under dense coding, we removed the 10% of KCs with the highest lifetime sparseness or the 5% of KCs with the highest valence specificity (fractions correspond to the approximate parts of the cdfs where the random model had higher values) (dashed horizontal lines in Fig. 3 *C* and *D*) and replaced them with useless KCs (either silent or responding equally to all odors to preserve the 0.9 coding level). Indeed, in these cases, the random model no longer outperformed the homogeneous model (Fig. 3*F*). However, these changes did not correspond to the effects of removing the sparsest or most specific KCs on angular distance or dimensionality (*SI Appendix*, Fig. S2*I*), again indicating that angular distance and dimensionality do not always correspond to performance in our model.

Together, these results indicate that under sparse (but not dense) coding, introducing realistic inter-KC variability in *w*, *N*, and *θ* worsens the performance of the network by making KCs’ odor response profiles less consistently sparse and thus less specific to rewarded/punished odors. Because the real mushroom body uses sparse coding, we focus the rest of our analysis on the sparse coding condition (coding level = 0.1).

### Compensatory Tuning of KC Parameters Rescues Memory Performance

Because the central problem for memory performance in the random model was inter-KC variability in activity, we hypothesized that performance could be rescued in models where KCs could achieve roughly equal activity across the population while still preserving experimentally realistic variability in spiking thresholds and number/strength of excitatory inputs.

#### Activity-independent tuning of excitatory input weights

**Fig. 4.**
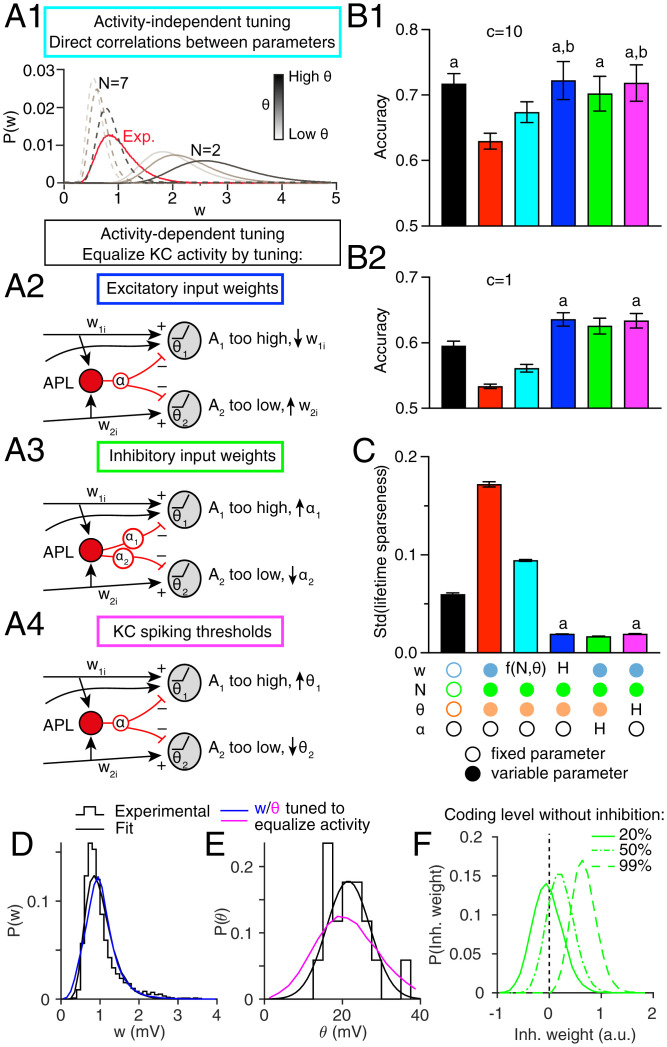
Compensation in network parameters rescues memory performance. (*A*) Schematics of different compensation methods. (*A*1) Activity-independent compensation. Log-normal fit of experimental distribution of the synaptic weights (Exp.; red) and its component distributions for different N and *θ* for high N = 7 (dashed) or low N=2 (solid). Shades of gray indicate different values of *θ*. (*A*2*–A*4) Mechanisms for activity-dependent homeostatic compensation. Overly active KCs weaken excitatory input weights (*w_ji_*; *A*2), strengthen inhibitory input weights (*α_j_*; *A*3), or raise spiking thresholds (*θ_j_*; *A*4). Inactive KCs do the reverse. (*B*1) Compensation rescues performance, alleviating the defect caused by inter-KC variability in the random model (red) compared with the homogeneous model (black) whether compensation occurs by setting w according to N and *θ* (cyan; *A*1) or using activity-dependent homeostatic compensation to adjust excitatory weights (blue; *A*2), inhibitory weights (green; *A*3), or spiking thresholds (magenta; *A*4). (*B*2) Differences between models are more apparent when the task is more difficult due to more stochastic decision making (*c* = 1 instead of *c* = 10 in the softmax function). (*C*) Compensation reduces variability in KC lifetime sparseness. *n* = 20 model instances with different random PN–KC connectivity; error bars are 95% CIs. All bars are significantly different from each other unless they share the same letter annotations; *P* < 0.05 by Wilcoxon signed rank test (for matched models with the same PN–KC connectivity) or Mann–Whitney test (for unmatched models with different PN–KC connectivity; i.e., fixed vs. variable N), with Holm–Bonferroni correction for multiple comparisons (full statistics are in Dataset S1). Annotations below bars indicate whether parameters were fixed (empty circles), variable (filled circles), or variable following a compensation rule [“H” for homeostatic tuning; f(N,θ) for activity-independent tuning]. Results here are for 100 synthetic odors; *SI Appendix*, Fig. S1*B* shows similar results with odors from ref. 30. (*D*) KC excitatory input synaptic weights (w) after tuning to equalize average activity (blue) follow a similar distribution to experimental data (black) (from Fig. 2*A*1). (*E*) KC spiking thresholds (*θ*) after tuning to equalize average activity (magenta) have wider variability than the experimental distribution (black) (from Fig. 2*A*3). (*F*) Tuning KC inhibitory weights (*α*) to equalize average activity requires many inhibitory weights to be negative, unless the coding level without inhibition is as high as 99%.

First, we tested a model that equalizes KC activity indirectly by making parameters compensate for each other in an activity-independent way. In particular, we modeled KCs as adjusting input synaptic weights (*w*) to compensate for variability in spiking threshold (*θ*) and number of PN inputs (*N*). Thus, an individual KC with low *θ* or high *N* would have low *w*, while a KC with high *θ* or low *N* would have high *w*. We simulated these correlations ($w\propto \sqrt{\theta }$; $w\propto 1/\sqrt{N}$) constrained by experimental data. To do this, we sampled *N* and *θ* from the distributions in Fig. 2*A* and sampled *w* from a posterior compensatory distribution, P(w|N,θ), whose overall shape across all KCs was constrained to be the same as the experimental *P*(*w*) in Fig. 2*A*1 but which was composed of multiple distributions of *P*(*w*) for different values of *N* and *θ*. For example, a KC with a relatively high *N* = 7 would sample its weights from a *P*(*w*) shifted to the left (lower *w*) (Fig. 4*A*1, dashed lines), while a KC with a relatively low *N* = 2 would sample its weights from a *P*(*w*) shifted to the right (higher *w*) (Fig. 4*A*1, solid lines). The same would be true for different values of *θ* (Fig. 4*A*1, different shadings). We fitted these component *P*(*w*) curves so that with experimentally observed distributions of *N* and *θ*, the sum of the components would produce the experimentally observed distribution of *w* across all KCs (*SI Appendix*). (Note that this algorithm is not meant to describe an actual biological mechanism, merely to create correlations between *w* vs. *N* and *θ* while constraining the parameters to experimentally realistic distributions. Biologically, such correlations could arise through several mechanisms [*Discussion*].) This compensatory mechanism rescued the fly’s performance, producing significantly higher accuracy at classifying odors than the random model (cyan bars in Fig. 4*B* and *SI Appendix*, Fig. S1*B*), likely resulting from the reduced variability in KC lifetime sparseness (Fig. 4*C*). (Note, however, that this model did not perform quite as well as the homogeneous model.)

#### Activity-dependent tuning of KC parameters

We next tested compensatory mechanisms based on activity rather than explicit correlations between network parameters. Here, each KC has the same desired average activity level across all odors, *A*_0_ (with a tolerance of ±6%). We tested three models, each of which equalized average KC activity *A*_0_ by tuning a different parameter: input excitatory weights (*w*), inhibitory weights (*α*), or spiking thresholds (*θ*). The nontuned parameters followed the distributions in Fig. 2*A* (inhibitory weights were constant when nontuned), while individual KCs adjusted the tuned parameter according to whether their activity was too high or too low. For example, a relatively highly active KC (whether because it has high *w* or *N*, was low *θ*, or simply receives input from highly active PNs) would scale down its excitatory weights (Fig. 4*A*2), scale up its inhibitory weights (Fig. 4*A*3), or scale up its spiking threshold (Fig. 4*A*4). Likewise, a relatively inactive (or indeed, totally silent) KC would do the reverse (details of the update rules underlying the homeostatic tuning and discussion of variant update rules are in *SI Appendix*, Figs. S3 and S4).

All three homeostatic models performed as well as the homogeneous model (blue, green, and magenta bars in Fig. 4*B*1 and *SI Appendix*, Fig. S1*B*) and indeed, even outperformed the homogeneous model when decision making was more stochastic (lower value of *c* in the softmax function) (Fig. 4*B*2). The more stochastic decision making makes the task more difficult and thus, brings out the enhanced coding by the homeostatic models. Indeed, the variability in KC lifetime sparseness was even lower in the homeostatic models than in the homogeneous model (Fig. 4*C*). (As average activity and lifetime sparseness are not the same thing, it is notable that tuning to equalize average activity also tended to equalize lifetime sparseness.)

What distributions of excitatory weights, inhibitory weights, or spiking thresholds emerge after activity-dependent tuning to equalize KC activity? Do they match experimentally observed distributions? Tuning excitatory weights led to a distribution fairly similar to the approximately log-normal experimentally observed distribution of amplitudes of excitatory postsynaptic potentials (EPSPs) (Fig. 4*D*). Tuning spiking thresholds led to a distribution with greater variance than the experimental distribution, although with a qualitatively similar Gaussian shape (Fig. 4*E*). This larger variance of thresholds suggests that natural variation of *θ* is too small, on its own, to equalize KC activity given the variation in the number/strength of excitatory inputs.

The tuned distribution of inhibitory weights differed even more strongly from experimental results. While there are no experimental measurements of inhibitory weights, equalizing KC activity by tuning inhibitory weights required many of them to be negative (Fig. 4*F*), which is unrealistic, because negative inhibition is actually excitation, and there are no reports of KCs being excited by *γ*-aminobutyric acid (41). Our model required negative inhibition because of the constraint that inhibition is only strong enough to reduce the fraction of active KCs by half (from 20 to 10%, based on results from ref. 25). In other words, 80% of the time, KCs are silent even without inhibition, thanks to high thresholds; such responses cannot be increased by reducing inhibition unless inhibition becomes negative (i.e., excitatory). Indeed, if we relax the constraint that the coding level be 0.2 without inhibition, such that sparseness is enforced by inhibition alone (not thresholds), then variable inhibition can equalize KC activity without becoming negative (Fig. 4*F*). However, in this case, the coding level without inhibition was 99%, which is not observed experimentally (25). Even allowing a coding level without inhibition of 50%, equalizing KC activity still requires some APL–KC inputs to be negative (Fig. 4*F*). Interestingly, these unrealistic models, where sparseness is mainly driven by inhibition rather than high thresholds, perform better than the three models shown here (*SI Appendix*, Fig. S4*A*), suggesting that biological constraints may limit network performance. Overall, these results suggest that tuning inhibitory weights cannot compensate on its own for variability in other KC parameters. More likely, the system optimizes multiple parameters at once (*Discussion*; see Fig. 6).

We also tested whether memory performance can be rescued by equalizing not KC average activity but rather, KC response probability (equivalent to average activity if KC activity is binarized; i.e., zero or one). Equalizing response probability (as opposed to average activity) by tuning KC spiking thresholds has been shown to improve separation of KC odor representations in a different computational model (34). However, in our model, this technique (tuning thresholds to equalize KC response probability) produced somewhat worse classification performance compared with tuning thresholds to equalize KC average activity (*SI Appendix*, Fig. S4 *B*1, *B*2, and *C*), although still better than the random model (compare Fig. 4 with *SI Appendix*, Fig. S4).

### Robustness of Pretuned Compensations in New Environments with Novel Odors

Any activity-dependent tuning depends on the model’s context. If a fly tunes its network parameters based on experience in one odor context (e.g., smelling only odors of one chemical family), will it still perform well at classifying odors in a novel environment with different odors (e.g., odors of a different chemical family)? We hypothesized that performance would depend more on tuning context with the activity-dependent compensation mechanisms than the activity-independent mechanism.

**Fig. 5.**
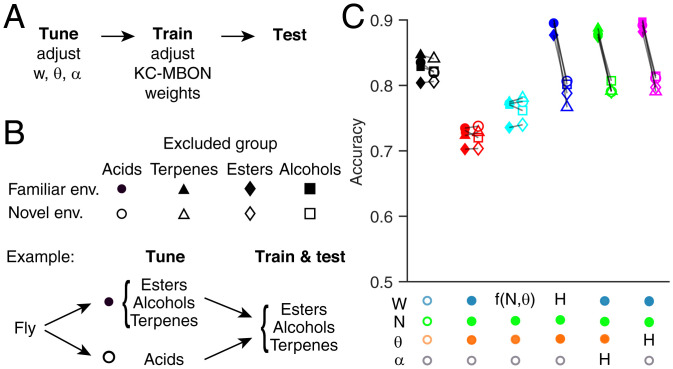
Robustness of pretuned compensations with novel odors. (*A*) For each model fly, network parameters are tuned as in Fig. 4 on a subset of odors. At this stage, no rewards or punishments are given, and KC output weights are not modified. Then, the model is trained to classify rewarded and punished odors that are the same as or different from the odors used for tuning. Finally, the model is tested on new noisy variants of the odors used for training. (*B*) Empty symbols (novel environment): models were tuned on odors from one chemical group (*G_i_*: acids, circles; terpenes, triangles; esters, diamonds; or alcohols, squares), and then, they were trained and tested on odors from the other three groups ($G_{i\neq j}$). Each empty symbol is paired with a matched control (filled symbols) showing how that model would have fared in a familiar environment (i.e., a model tuned, trained, and tested all on the same three groups of odors that the matched novel model was trained and tested on [$G_{i\neq j}$]). (*C*) Models with activity-dependent compensation (blue, magenta, and green) performed significantly worse in the novel environment than familiar environments (matching indicated by connecting lines; *P* < 0.05, Wilcoxon signed rank test with Holm–Bonferroni correction). In contrast, models with no compensation (black and red) or activity-independent compensation (cyan) performed similarly in novel and familiar environments (*P* > 0.05 except for homogeneous [black] acids and random [red] terpenes) (full statistics in Dataset S1). Mean of 20 model instantiations, where each instantiation received a different permutation of odors (*SI Appendix*). Annotations below the graph indicate whether parameters were fixed (empty circles), variable (filled circles), or variable following a compensation rule [H for homeostatic tuning, f(N, θ) for activity-independent tuning].

To test this, we tuned the parameters in our models using only a subset of odors from ref. 30, grouped by chemical class, and then trained and tested the models on odor–reward/punishment associations using the other odors. We took the four chemical classes that had the most odors in the dataset: acids, terpenes, alcohols, and esters. For each class, we tuned the model’s parameters on that class and then trained the model to classify odors in the other three classes (“novel” environment). For matched controls, we trained models that had been tuned on the same three classes used for training/testing (“familiar” environment). As expected, the three activity-dependent models performed worse in novel environments than familiar environments, while the activity-independent model performed consistently regardless of tuning environment (blue, green, and magenta vs. cyan in Fig. 5*C*). However, in general, tuning odors on one class but training/testing on different classes does not fatally damage the activity-dependent compensation strategies; although performance is worse in novel environments, it remains better than the random model. Thus, activity-dependent compensation is still a good strategy to overcome the pernicious effects of inter-KC variation, even if the compensation environment differs from the classification environment (at least within the range of the odors in ref. 30).

### Connectome Reveals Compensatory Variation of Input Strength and Numbers

**Fig. 6.**
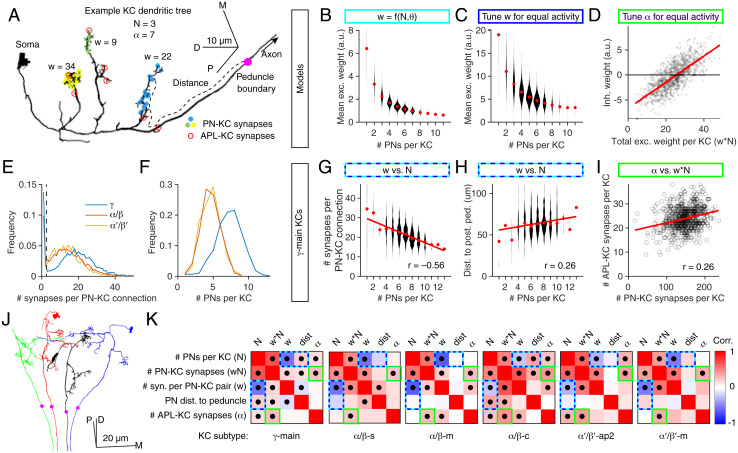
Connectome analysis reveals compensatory variation in excitatory and inhibitory input strengths. (*A*) Example αβ-c KC (body identification 5901207528) with inputs from three PNs (yellow, green, and blue dots) and seven dendritic APL–KC synapses (red circles). The magenta circle shows the posterior boundary of the peduncle. Line widths are not to scale. (*B* and *C*) Mean synaptic weight (w) per PN–KC connection is inversely related to the number of input PNs in models that tune input weights given N and *θ* (*B*) or that tune input weights to equalize average activity levels across KCs (*C*). (*D*) In the model that tunes input inhibitory synaptic weights (*α*) to equalize average activity levels across KCs, inhibitory weights are directly related to the sum of excitatory weights per KC (i.e., wN). Note the negative values of *α* (discussed in the text). (*E* and *F*) Probability distributions of the number of synapses per PN–KC connection (*E*) and the number of input PNs per KC (*F*) in the different KCs subtypes (αβ,γ,α′β′). The dashed line in *E* shows our threshold for counting connections as genuine. (*G*) Mean number of input synapses per PN–KC connection (averaged across PNs for each KC) is inversely related to the number of input PNs per KC in *γ*-main KCs (*SI Appendix*, Fig. S5 shows other KC types). (*H*) Mean distance of PN–KC synapses to the posterior boundary of the peduncle (presumed spike initiation zone) is directly related to the number of input PNs per KC. (*I*) The number of APL–KC synapses per KC is directly related to the total number of PN–KC synapses per KC. (*J*) Four αβ-c KCs, one from each neuroblast clone. The posterior boundary of the peduncle (magenta circles) lies where the KC axons begin to converge. (*K*) Grids show Pearson correlation coefficients (*r*) between various KC parameters for all KC subtypes tested (red, positive; blue, negative). Dots indicate *P* < 0.05 (Holm–Bonferroni corrected) (full statistics are in Dataset S1). Colored outlines indicate predictions of models (cyan/blue, models tuning w [*G* and *H*]; green, model tuning *α* [*I*]). Number of KCs for each subtype, from left to right, are 588, 222, 350, 220, 127, and 119. In *B*, *C*, *G*, and *H*, red dots are medians, and the widths of the violin plots represent the number of KCs in each bin. Trend lines in *D*, *G*, *H*, and *I* show linear fits to the data. D, dorsal; M, medial; P, posterior.

Our proposed compensatory mechanisms predict correlations between the key model parameters. Excitatory weights (*w*) should be inversely correlated to number of PNs per KC (*N*), where *w* is tuned to compensate for variable *N* and *θ* (Fig. 6*B*) or where *w* is tuned to equalize KC activity (Fig. 6*C*). Meanwhile, inhibitory weights (*α*) should be positively correlated to the sum of excitatory weights (∑ *w* or $\bar{w}N$, where $\bar{w}$ is the mean *w* per KC), where inhibitory weights are tuned to equalize KC activity (Fig. 6*D*). Such correlations have been observed in larvae (42), but they have not yet been analyzed in the adult mushroom body.

To test these predictions, we analyzed the recently published hemibrain connectome (43, 44), which annotates all synapses between PNs and KCs in the right mushroom body of one fly. The connectome reveals three of our parameters: the number of PN inputs per KC (*N*), the strength of each PN–KC connection (*w*), and the strength of inhibitory inputs (*α*). Although the anatomy does not directly reveal *w* and *α* (which can only be measured electrophysiologically), we used an indirect proxy for synaptic strength: the number of synapses per connection (i.e., number of sites between two neurons where neuron 1 has a T bar and neuron 2 has a postsynaptic density, counted by machine vision) (Fig. 6*A*). It seems reasonable to presume that, all else being equal, connections with more synapses are stronger. Indeed, in the *Drosophila* antennal lobe, when comparing connections from ORNs with ipsilateral PNs vs. contralateral PNs, ipsilateral connections are both stronger (45) and have more synapses per connection (46). Moreover, synaptic counts approximate synaptic contact area throughout the larval *Drosophila* nervous system (47), and synaptic area approximates EPSP amplitude in mammalian cortex (48).

Therefore, to test if mean *w* and *N* are inversely correlated across KCs, we asked if the number of PN inputs per KC was inversely correlated to the number of synapses per PN–KC connection. We ignored PN–KC connections with two or fewer synapses because the number of synapses per PN–KC connection formed a bimodal distribution with a trough around three to four (Fig. 6*E*); we presumed that connections with only one to two synapses represent annotation errors. We divided KCs into their different subtypes as annotated in the hemibrain (44) because different subtypes have different numbers of PN inputs per KC and different numbers of synapses per PN–KC connection (28) (Fig. 6 *E* and *F* and *SI Appendix*, Fig. S5). We excluded KCs that receive significant nonolfactory input (γ-d,γ-t,αβ-p,α′β′-ap1). In all analyzed subtypes of KCs (*γ*-main; αβ-s, -m, and -c; α′β′-ap2 and -m), the number of PN inputs per KC (*N*) was inversely correlated to the mean number of synapses per PN–KC connection, averaged across the PN inputs onto a KC (proxy for $\bar{w}$) (Fig. 6 *G* and *K* and *SI Appendix*, Fig. S5). Linear regression showed that, on average, there were ≈6−15% fewer input synapses per PN–KC connection ($\bar{w}$) for each additional PN per KC (*N*) (compare with the equivalent slopes for the linear fits to the activity-independent [–22%] and activity-dependent [–18%] model parameters in Fig. 6 *B* and *C*). This negative correlation meant that the number of total PN–KC synapses per KC increased only sublinearly relative to the number of PN inputs per KC (*SI Appendix*, Fig. S5).

We also tested another anatomical proxy of excitatory synaptic strength. Because KCs sum up synaptic inputs linearly or sublinearly, their dendrites likely lack voltage-gated currents that would amplify inputs, so synaptic input currents likely propagate passively (26). Therefore, an excitatory input would make a smaller contribution to a KC’s decision to spike the farther away it is from the spike initiation zone (49). While the spike initiation zone cannot be directly observed in the connectome, the voltage-gated Na^+^ channel Para and other markers of the axon initial segment (also called the “distal axonal segment”) are concentrated at the posterior end of the peduncle, near where axons from KCs derived from the four neuroblast clones converge (50, 51). This location can be approximated in the connectome as the posterior boundary of the “PED(R)” (i.e., peduncle) region of interest (ROI) (magenta dots in Fig. 6 *A* and *J*). From this point, we measured the distance along each KC’s neurite skeleton (i.e., not the Euclidean distance) to each PN–KC synapse. In the αβ-c and *γ*-main KCs (but not other KCs), this distance was positively correlated with the number of PNs per KC (Fig. 6 *H* and *K* and *SI Appendix*, Fig. S5). That is, the more PN inputs a KC has, the farther away the input synapses are from the putative spike initiation zone (and thus, the weaker they are likely to be). Intriguingly, of all the KC subtypes, αβ-c KCs show the strongest correlation between number of PN inputs and PN–peduncle distance but the weakest correlation between number of PN inputs and number of synapses per PN–KC connection (Fig. 6*K*), suggesting that different types of KCs might use different mechanisms to achieve the same compensatory end.

To test if inhibitory and excitatory input are positively correlated across KCs (as predicted in Fig. 6*D*), we approximated *α* by counting the number of synapses from the APL neuron to every KC in the calyx (annotated as the “CA(R)” ROI in the connectome). In all types of KCs, the more total PN–KC synapses there were per KC, the more calyx APL–KC synapses there were (Fig. 6 *I* and *K* and *SI Appendix*, Fig. S5), indicating that, indeed, inhibitory and excitatory synaptic inputs are correlated.

These results confirm the predictions of our compensatory models. That correlations exist for both excitation and inhibition suggests that the mushroom body tunes more than one parameter simultaneously (thresholds may be tuned as well but cannot be measured in the connectome). Such multiparameter optimization likely explains 1) why the correlations in the connectome are not as steep as when only a single parameter is tuned in our models (Fig. 6 *D–F*) and 2) why natural compensatory variation of tuned parameters need not be as wide as the variation of tuned parameters in our models (Fig. 4*E*).

## Discussion

Here, we studied under what conditions interneuronal variability would improve vs. impair associative memory. Using a computational model of the fly mushroom body, we showed that under sparse coding conditions, associative memory performance is reduced by experimentally realistic variability among KCs in parameters that control neuronal excitability (spiking threshold and the number/strength of excitatory inputs). These deficits arise from unequal average activity levels among KCs. However, memory performance can be rescued by using variability along one parameter to compensate for variability along other parameters, thereby equalizing average activity among KCs. These compensatory models predicted that certain KC features would be correlated with each other, and these predictions were borne out in the hemibrain connectome. In short, we showed 1) the computational benefits of compensatory variation, 2) multiple mechanisms by which such compensation can occur, and 3) anatomical evidence that such compensation does, in fact, occur.

Note that when we say “equalizing KC activity,” we do not mean that all KCs should respond the same to a given odor. Rather, in each responding uniquely to different odors (due to their unique combinations of inputs from different PNs), they should keep their average activity levels the same. That is, while KCs’ odor responses should be heterogeneous, their average activity should be homogeneous.

How robust are our connectome analyses? We found correlations between anatomical proxies for the physiological properties predicted to be correlated in our models (i.e., KCs receiving excitation from more PNs should have weaker excitatory inputs, while KCs receiving more overall excitation should also receive more inhibition). In particular, we measured the number of synapses per connection as a proxy for the strength of a connection. As described above, this proxy seems valid based on matching anatomical and electrophysiological data (46–48). However, other factors affecting synaptic strength (receptor expression, posttranslational modification of receptors, presynaptic vesicle release, input resistance, etc.) would not be visible in the connectome. Of course, such factors could further enable compensatory variability (see below). It is also worth noting that the connectome data are from only one individual.

We also used the distance between PN–KC synapses and the peduncle as a proxy for the passive decay of synaptic currents as they travel to the spike initiation zone. In the absence of detailed compartmental models of KCs, it is hard to predict exactly how much increased distance would reduce the effective strength of synaptic inputs, but it is plausible to assume that signals decay monotonically with distance. Note that calcium signals are often entirely restricted to one dendritic claw (26, 52). Another caveat is that the posterior boundary of the peduncle is only an estimate [although a plausible one (50, 51)] of the location of the spike initiation zone. However, inaccurate locations should only produce fictitious correlations for Fig. 6*J* and *SI Appendix*, Fig. S5*F* if the error is correlated with the number of PN–KC synapses per KC (and only in αβ-c and *γ*-main KCs, not other KCs), which seems unlikely.

Our work is consistent with prior work, both theoretical and experimental, showing that compensatory variability can maintain consistent network behavior (1–11, 53, 54). However, here we analyze the computational benefits of equalizing activity levels across neurons in a population (as opposed to across individual animals or over time). A recent preprint showed that equalizing response probabilities among KCs reduces memory generalization (34), but we showed that equalizing average activity outperforms equalizing response probabilities (*SI Appendix*, Fig. S4). Another model of the mushroom body used compensatory inhibition, in which the strength of inhibition onto each KC was proportional to its average excitation (31), similar to our inhibitory plasticity model (Fig. 4*A*2). However, the previous work did not analyze the specific benefits from the compensatory variation; it also set the inhibition strong enough that average net excitation was zero, whereas we show that when inhibition is constrained to be only strong enough to reduce KC activity by approximately half [consistent with experimental data (25)], inhibition alone cannot realistically equalize KC activity (Fig. 4*G*). In addition, there is experimental support for our models’ predictions that KCs with more PN inputs would have weaker excitatory inputs; when predicting whether calcium influxes in individual claws would add up to cause a suprathreshold response in the whole KC, the most accurate prediction came from dividing the sum of claw responses by the log of the number of claws (52). However, the functional benefits of this result only become clear with our computational models. Finally, the larval mushroom body shows a similar relationship between number and strength of PN–KC connections; the more PN inputs a KC has, the fewer synapses per PN–KC connection (42); however, again, the larval work did not analyze the computational benefits of this correlation.

We modeled two forms of compensation: direct correlations between neuronal parameters (Fig. 4*A*1) and activity-dependent homeostasis (Fig. 4*A*2*–A*4). Both forms improve performance and predict observed correlations in the connectome. Certainly, activity-dependent mechanisms are plausible as KCs regulate their own activity homeostatically in response to perturbations in activity (55). Indeed, different KC subtypes use different combinations of mechanisms for homeostatic plasticity (55), consistent with the different correlations observed in the connectome for different KC subtypes. Our activity-dependent models lend themselves to straightforward biological interpretations. Excitatory or inhibitory synaptic weights could be tuned by activity-dependent regulation of the number of synapses per connection or expression/localization of receptors or other postsynaptic machinery. Spiking thresholds could be tuned by altering voltage-gated ion conductances or moving/resizing the spike initiation zone (51, 56). Such homeostatic plasticity would be akin to the sensory gain control implemented by feedback inhibition but on a slower timescale.

On the other hand, KCs are not infinitely flexible in homeostatic regulation; for example, complete blockade of inhibition causes the same increase in KC activity regardless of whether the blockade is acute (16 to 24 h) or constitutive (throughout life) (55). This apparent lack of activity-dependent down-regulation of excitation suggests that activity-independent mechanisms might contribute to compensatory variation in KCs, as occurs for ion conductances in lobster stomatogastric ganglion neurons (8, 9). For example, the inverse correlation of *w* and *N* arises from the fact that the number of PN–KC synapses per KC increases only sublinearly with increasing numbers of claws (i.e., PN inputs) (*SI Appendix*, Fig. S5*H*). Perhaps a metabolic or gene regulatory constraint prevents claws from recruiting postsynaptic machinery in linear proportion to their number. [Interestingly, this suppression is stronger in larvae, where the number of PN–KC synapses per KC is actually constant relative to the number of claws (42).] Meanwhile, the correlation between the number of inhibitory synapses and the number of excitatory synapses might be explained if excitatory and inhibitory synapses share bottleneck synaptogenesis regulators on the postsynaptic side. Although activity-dependent compensation produced superior performance in our model compared with activity-independent compensation thanks to its more effective equalization of KC average activity (Fig. 4) (most likely because it takes into account the unequal activity of different PNs), activity-dependent mechanisms suffered when the model network switched to a novel odor environment (Fig. 5). Given that it is desirable for even a newly eclosed fly to learn well and for flies to learn to discriminate arbitrary novel odors, activity-independent mechanisms for compensatory variation may be more effective in nature.

Compensatory variability to equalize activity across neurons could also occur in other systems. The vertebrate cerebellum has an analogous architecture to the insect mushroom body; cerebellar granule cells are strikingly similar to KCs in their circuit anatomy, proposed role in “expansion recoding” for improved memory, and even signaling pathways for synaptic plasticity (21, 39, 57–60). Whereas cortical neurons’ average spontaneous firing rates vary over several orders of magnitude (61), granule cells are, like KCs, mostly silent at rest, and it is plausible that their average activity levels might be similar (while maintaining distinct responses to different stimuli) (62). Granule cell input synapses undergo homeostatic plasticity (63), while compartmental models suggest that differences in granule cells’ dendritic morphology would affect their activity levels, an effect attenuated by inhibition (64), raising the possibility that granule cells may also modulate interneuronal variability through activity-dependent mechanisms. Future experiments may test whether compensatory variability occurs in, and improves the function of, the cerebellum or other brain circuits. Finally, activity-dependent compensation may provide useful techniques for machine learning. For example, we found that performance of a reservoir computing network could be improved if thresholds of individual neurons are initialized to achieve a particular activity probability given the distribution of input activities (65).

## Materials and Methods

Full details of the computational models are given in *SI Appendix*. For Fig. 6, KC neurite skeletons and connectivity were downloaded from the hemibrain connectome v. 1.1 (43). KCs with truncated skeletons lacking the dendritic tree were excluded. The distance from each PN–KC synapse to the posterior boundary of the peduncle along the KC’s neurite skeleton (i.e., not the Euclidean distance) was measured as described in ref. 36. *SI Appendix* has further details. Modeling and connectome analysis were carried out using custom code written in MATLAB, which is available at https://github.com/aclinlab/CompensatoryVariability.

## Supplementary Material

Supplementary File

Supplementary File

## Data Availability

Code has been deposited in GitHub (https://github.com/aclinlab/CompensatoryVariability) and the raw simulation results are in Dataset S1. Previously published data were also used for this work [Scheffer et al. (43)].

## References

[1] J. Golowasch, M. S. Goldman, L. F. Abbott, E. Marder, Failure of averaging in the construction of a conductance-based neuron model. J. Neurophysiol. 87, 1129–1131 (2002). 11826077 10.1152/jn.00412.2001

[2] P. Achard, E. De Schutter, Complex parameter landscape for a complex neuron model. PLoS Comput. Biol. 2, e94 (2006). 16848639 10.1371/journal.pcbi.0020094 PMC1513272

[3] A. E. Tobin, R. L. Calabrese, Endogenous and half-center bursting in morphologically-inspired models of leech heart interneurons. J. Neurophysiol. 96, 2089–2106 (2006). 16760353 10.1152/jn.00025.2006 PMC2902779

[4] A. L. Taylor, J. M. Goaillard, E. Marder, How multiple conductances determine electrophysiological properties in a multicompartment model. J. Neurosci. 29, 5573–5586 (2009). 19403824 10.1523/JNEUROSCI.4438-08.2009 PMC2821064

[5] E. Marder, J. M. Goaillard, Variability, compensation and homeostasis in neuron and network function. Nat. Rev. Neurosci. 7, 563–574 (2006). 16791145 10.1038/nrn1949

[6] D. J. Schulz, J. M. Goaillard, E. Marder, Variable channel expression in identified single and electrically coupled neurons in different animals. Nat. Neurosci. 9, 356–362 (2006). 16444270 10.1038/nn1639

[7] D. J. Schulz, J. M. Goaillard, E. E. Marder, Quantitative expression profiling of identified neurons reveals cell-specific constraints on highly variable levels of gene expression. Proc. Natl. Acad. Sci. U.S.A. 104, 13187–13191 (2007). 17652510 10.1073/pnas.0705827104 PMC1933263

[8] J. N. MacLean, Y. Zhang, B. R. Johnson, R. M. Harris-Warrick, Activity-independent homeostasis in rhythmically active neurons. Neuron 37, 109–120 (2003). 12526777 10.1016/s0896-6273(02)01104-2

[9] J. N. MacLean ., Activity-independent coregulation of IA and Ih in rhythmically active neurons. J. Neurophysiol. 94, 3601–3617 (2005). 16049145 10.1152/jn.00281.2005

[10] T. O’Leary, E. Marder. Temperature-robust neural function from activity-dependent ion channel regulation. Curr Biol. 26, 2935–2941 (2016). 27746024 10.1016/j.cub.2016.08.061 PMC5111818

[11] J. Z. Parrish ., Krüppel mediates the selective rebalancing of ion channel expression. Neuron 82, 537–544 (2014). 24811378 10.1016/j.neuron.2014.03.015 PMC4104505

[12] J. Gjorgjieva, G. Drion, E. Marder, Computational implications of biophysical diversity and multiple timescales in neurons and synapses for circuit performance. Curr. Opin. Neurobiol. 37, 44–52 (2016). 26774694 10.1016/j.conb.2015.12.008 PMC4860045

[13] G. Marsat, L. Maler, Neural heterogeneity and efficient population codes for communication signals. J. Neurophysiol. 104, 2543–2555 (2010). 20631220 10.1152/jn.00256.2010

[14] F. Zeldenrust, B. Gutkin, S. Denéve, Efficient and robust coding in heterogeneous recurrent networks. PLoS Comput. Biol. 17, e1008673 (2021). 33930016 10.1371/journal.pcbi.1008673 PMC8115785

[15] K. Padmanabhan, N. N. Urban, Intrinsic biophysical diversity decorrelates neuronal firing while increasing information content. Nat. Neurosci. 13, 1276–1282 (2010). 20802489 10.1038/nn.2630 PMC2975253

[16] K. Padmanabhan, N. N. Urban, Disrupting information coding via block of 4-AP-sensitive potassium channels. J. Neurophysiol. 112, 1054–1066 (2014). 24899672 10.1152/jn.00823.2013 PMC4122725

[17] S. J. Tripathy, K. Padmanabhan, R. C. Gerkin, N. N. Urban, Intermediate intrinsic diversity enhances neural population coding. Proc. Natl. Acad. Sci. U.S.A. 110, 8248–8253 (2013). 23630284 10.1073/pnas.1221214110 PMC3657795

[18] L. Manneschi ., Exploiting multiple timescales in hierarchical echo state networks. Front. Appl. Math. Stat. 6, 616658 (2021).

[19] N. Perez-Nieves, V. C. H. Leung, P. L. Dragotti, D. F. M. Goodman, Neural heterogeneity promotes robust learning. Nat. Commun. 12, 5791 (2021). 34608134 10.1038/s41467-021-26022-3 PMC8490404

[20] D. Owald ., Activity of defined mushroom body output neurons underlies learned olfactory behavior in *Drosophila*. Neuron 86, 417–427 (2015). 25864636 10.1016/j.neuron.2015.03.025 PMC4416108

[21] A. Handler ., Distinct dopamine receptor pathways underlie the temporal sensitivity of associative learning. Cell 178, 60–75.e19 (2019). 31230716 10.1016/j.cell.2019.05.040 PMC9012144

[22] T. Hige, Y. Aso, M. N. Modi, G. M. Rubin, G. C. Turner, Heterosynaptic plasticity underlies aversive olfactory learning in *Drosophila*. Neuron 88, 985–998 (2015). 26637800 10.1016/j.neuron.2015.11.003 PMC4674068

[23] H. Amin, A. C. Lin, Neuronal mechanisms underlying innate and learned olfactory processing in *Drosophila*. Curr. Opin. Insect Sci. 36, 9–17 (2019). 31280185 10.1016/j.cois.2019.06.003

[24] K. S. Honegger, R. A. A. Campbell, G. C. Turner, Cellular-resolution population imaging reveals robust sparse coding in the *Drosophila* mushroom body. J. Neurosci. 31, 11772–11785 (2011). 21849538 10.1523/JNEUROSCI.1099-11.2011 PMC3180869

[25] A. C. Lin, A. M. Bygrave, A. de Calignon, T. Lee, G. Miesenböck, Sparse, decorrelated odor coding in the mushroom body enhances learned odor discrimination. Nat. Neurosci. 17, 559–568 (2014). 24561998 10.1038/nn.3660 PMC4000970

[26] E. Gruntman, G. C. Turner, Integration of the olfactory code across dendritic claws of single mushroom body neurons. Nat. Neurosci. 16, 1821–1829 (2013). 24141312 10.1038/nn.3547 PMC3908930

[27] G. C. Turner, M. Bazhenov, G. Laurent, Olfactory representations by *Drosophila* mushroom body neurons. J. Neurophysiol. 99, 734–746 (2008). 18094099 10.1152/jn.01283.2007

[28] S. J. Caron, V. Ruta, L. F. Abbott, R. Axel, Random convergence of olfactory inputs in the *Drosophila* mushroom body. Nature 497, 113–117 (2013). 23615618 10.1038/nature12063 PMC4148081

[29] S. R. Olsen, V. Bhandawat, R. I. Wilson, Divisive normalization in olfactory population codes. Neuron 66, 287–299 (2010). 20435004 10.1016/j.neuron.2010.04.009 PMC2866644

[30] E. A. Hallem, J. R. Carlson, Coding of odors by a receptor repertoire. Cell 125, 143–160 (2006). 16615896 10.1016/j.cell.2006.01.050

[31] S. X. Luo, R. Axel, L. F. Abbott, Generating sparse and selective third-order responses in the olfactory system of the fly. Proc. Natl. Acad. Sci. U.S.A. 107, 10713–10718 (2010). 20498080 10.1073/pnas.1005635107 PMC2890779

[32] M. Parnas, A. C. Lin, W. Huetteroth, G. Miesenböck, Odor discrimination in *Drosophila*: From neural population codes to behavior. Neuron 79, 932–944 (2013). 24012006 10.1016/j.neuron.2013.08.006 PMC3765961

[33] K. Krishnamurthy, A. M. Hermundstad, T. Mora, Disorder and the neural representation of complex odors: Smelling in the real world. arXiv [Preprint] (2017). https://arxiv.org/abs/1707.01962 (Accessed 7 July 2017).

[34] A. Kennedy, Learning with naturalistic odor representations in a dynamic model of the *Drosophila* olfactory system. bioRxiv [Preprint] (2019). 10.1101/783191 (Accessed 11 October 2019).

[35] V. Bhandawat, S. R. Olsen, N. W. Gouwens, M. L. Schlief, R. I. Wilson, Sensory processing in the *Drosophila* antennal lobe increases reliability and separability of ensemble odor representations. Nat. Neurosci. 10, 1474–1482 (2007). 17922008 10.1038/nn1976 PMC2838615

[36] H. Amin, A. A. Apostolopoulou, R. Suárez-Grimalt, E. Vrontou, A. C. Lin, Localized inhibition in the *Drosophila* mushroom body. eLife 9, e56954 (2020). 32955437 10.7554/eLife.56954 PMC7541083

[37] Y. Aso, ., Mushroom body output neurons encode valence and guide memory-based action selection in *Drosophila*. eLife 3, e04580 (2014). 25535794 10.7554/eLife.04580 PMC4273436

[38] R. Cohn, I. Morantte, V. Ruta, Coordinated and compartmentalized neuromodulation shapes sensory processing in *Drosophila*. Cell 163, 1742–1755 (2015). 26687359 10.1016/j.cell.2015.11.019 PMC4732734

[39] A. Litwin-Kumar, K. D. Harris, R. Axel, H. Sompolinsky, L. F. Abbott, Optimal degrees of synaptic connectivity. Neuron 93, 1153–1164.e7 (2017). 28215558 10.1016/j.neuron.2017.01.030 PMC5379477

[40] B. Willmore, D. J. Tolhurst, Characterizing the sparseness of neural codes. Network 12, 255–270 (2001). 11563529

[41] K. Inada, Y. Tsuchimoto, H. Kazama, Origins of cell-type-specific olfactory processing in the *Drosophila* mushroom body circuit. Neuron 95, 357–367.e4 (2017). 28728024 10.1016/j.neuron.2017.06.039

[42] K. Eichler ., The complete connectome of a learning and memory centre in an insect brain. Nature 548, 175–182 (2017). 28796202 10.1038/nature23455 PMC5806122

[43] L. K. Scheffer ., A connectome and analysis of the adult Drosophila central brain. eLife 9, e57443 (2020). 32880371 10.7554/eLife.57443 PMC7546738

[44] F. Li ., The connectome of the adult Drosophila mushroom body provides insights into function. eLife 9, e62576 (2020). 33315010 10.7554/eLife.62576 PMC7909955

[45] Q. Gaudry, E. J. Hong, J. Kain, B. L. de Bivort, R. I. Wilson, Asymmetric neurotransmitter release enables rapid odour lateralization in *Drosophila*. Nature 493, 424–428 (2013). 23263180 10.1038/nature11747 PMC3590906

[46] W. F. Tobin, R. I. Wilson, W. A. Lee, Wiring variations that enable and constrain neural computation in a sensory microcircuit. eLife 6, e24838 (2017). 28530904 10.7554/eLife.24838 PMC5440167

[47] C. L. Barnes, D. Bonnery, A. Cardona, Synaptic counts approximate synaptic contact area in *Drosophila.* bioRxiv [Preprint] (2020). 10.1101/2020.10.09.333187 (Accessed 10 October 2020). PMC8979427 35377898

[48] S. Holler, G. Köstinger, K. A. C. Martin, G. F. P. Schuhknecht, K. J. Stratford, Structure and function of a neocortical synapse. Nature 591, 111–116 (2021). 33442056 10.1038/s41586-020-03134-2

[49] S. R. Williams, G. J. Stuart, Role of dendritic synapse location in the control of action potential output. Trends Neurosci. 26, 147–154 (2003). 12591217 10.1016/S0166-2236(03)00035-3

[50] T. A. Ravenscroft ., *Drosophila* voltage-gated sodium channels are only expressed in active neurons and are localized to distal axonal initial segment-like domains. J. Neurosci. 40, 7999–8024 (2020). 32928889 10.1523/JNEUROSCI.0142-20.2020 PMC7574647

[51] S. Trunova, B. Baek, E. Giniger, Cdk5 regulates the size of an axon initial segment-like compartment in mushroom body neurons of the *Drosophila* central brain. J. Neurosci. 31, 10451–10462 (2011). 21775591 10.1523/JNEUROSCI.0117-11.2011 PMC3150738

[52] H. Li, Y. Li, Z. Lei, K. Wang, A. Guo, Transformation of odor selectivity from projection neurons to single mushroom body neurons mapped with dual-color calcium imaging. Proc. Natl. Acad. Sci. 110, 12084–12089 (2013). 23818618 10.1073/pnas.1305857110 PMC3718165

[53] R. Grashow, T. Brookings, E. Marder, Compensation for variable intrinsic neuronal excitability by circuit-synaptic interactions. J. Neurosci. 30, 9145–9156 (2010). 20610748 10.1523/JNEUROSCI.0980-10.2010 PMC2913134

[54] H. Kazama, R. I. Wilson, Homeostatic matching and nonlinear amplification at identified central synapses. Neuron 58, 401–413 (2008). 18466750 10.1016/j.neuron.2008.02.030 PMC2429849

[55] A. A. Apostolopoulou, A. C. Lin, Mechanisms underlying homeostatic plasticity in the *Drosophila* mushroom body in vivo. Proc. Natl. Acad. Sci. U.S.A. 117, 16606–16615 (2020). 32601210 10.1073/pnas.1921294117 PMC7368247

[56] M. S. Grubb, J. Burrone, Activity-dependent relocation of the axon initial segment fine-tunes neuronal excitability. Nature 465, 1070–1074 (2010). 20543823 10.1038/nature09160 PMC3196626

[57] M. N. Modi, Y. Shuai, G. C. Turner, The *Drosophila* mushroom body: From architecture to algorithm in a learning circuit. Annu. Rev. Neurosci. 43, 465–484 (2020). 32283995 10.1146/annurev-neuro-080317-0621333

[58] S. M. Farris, Are mushroom bodies cerebellum-like structures? Arthropod Struct. Dev. 40, 368–379 (2011). 21371566 10.1016/j.asd.2011.02.004

[59] D. Marr, A theory of cerebellar cortex. J. Physiol. 202, 437–470 (1969). 5784296 10.1113/jphysiol.1969.sp008820 PMC1351491

[60] Y. Aso ., Nitric oxide acts as a cotransmitter in a subset of dopaminergic neurons to diversify memory dynamics. eLife 8, e49257 (2019). 31724947 10.7554/eLife.49257 PMC6948953

[61] G. Buzsáki, K. Mizuseki, The log-dynamic brain: How skewed distributions affect network operations. Nat. Rev. Neurosci. 15, 264–278 (2014). 24569488 10.1038/nrn3687 PMC4051294

[62] K. Powell, A. Mathy, I. Duguid, M. Häusser, Synaptic representation of locomotion in single cerebellar granule cells. eLife 4, 977 (2015). 10.7554/eLife.07290 PMC4499793 26083712

[63] I. Delvendahl, K. Kita, M. Müller, Rapid and sustained homeostatic control of presynaptic exocytosis at a central synapse. Proc. Natl. Acad. Sci. U.S.A. 116, 23783–23789 (2019). 31685637 10.1073/pnas.1909675116 PMC6876255

[64] C. M. Houston ., Exploring the significance of morphological diversity for cerebellar granule cell excitability. Sci. Rep. 7, 46147 (2017). 28406156 10.1038/srep46147 PMC5390267

[65] L. Manneschi, A. C. Lin, E. Vasilaki, SpaRCe: Improved learning of reservoir computing systems through sparse representations. IEEE Trans. Neural Netw. Learn. Syst., 10.1109/TNNLS.2021.3102378 (2021). 34398765

