# Supplementary material for: Compensatory variability in network parameters enhances memory performance in the Drosophila mushroom body
Source: Proc Natl Acad Sci U S A. 2021 Nov 29;118(49):e2102158118. doi: 10.1073/pnas.2102158118 (PMC8670477; doi:10.1073/pnas.2102158118)
Supplement: Supplementary File [file pnas.2102158118.sapp.pdf]

PNAS
www.pnas.org

Supplementary Information for

Compensatory variability in network parameters enhances memory performance in the *Drosophila* mushroom body

Nada Y. Abdelrahman, Eleni Vasilaki, Andrew C. Lin

For correspondence: Andrew Lin.
E-mail: andrew.lin@sheffield.ac.uk

This PDF file includes:

Supplementary text
Figs. S1 to S5
SI References

Supporting Information Text

Supplementary Methods

Modelling KC activity. PN activity was simulated using the odor responses of 24 olfactory receptors (1), passed through an equation proposed by (2). For an ORN and PN innervating the ith glomerulus, their responses to the kth odor can be described using ORN_i^k (ORN activity) and x_i^k (PN activity):

$$x_i^k = R_{max} \frac{(ORN_i^k)^{1.5}}{(ORN_i^k)^{1.5} + (s^k)^{1.5} + \sigma^{1.5}} \qquad [1]$$

where $s^k = m \sum_i ORN_i^k / 190$, $m = 10.63$, representing the gain of lateral inhibition in the antennal lobe, $R_{max} = 165$, representing the maximum PN response, and $\sigma = 12$, representing the non-linearity of the ORN-PN response function. We added noise to PN activity using:

$$(x_i^k)_{trial} = x_i^k (1 + CoV\mathcal{N}) \qquad [2]$$

where CoV is the coefficient of variation of PN activity across trials taken from Fig. 2E of (3) and $\mathcal{N}$ is a random sample drawn from a Gaussian distribution with mean 0 and standard deviation 1. For Fig. 2C3, the CoV was scaled by a factor of 0.5, 1 or 2. To increase the number of stimuli beyond the 110 recorded odors in (1), we generated odor responses in which the activity of each PN was randomly sampled from that PN's activity across the 110 odors used in (1), i.e., $x_i^k = x_i^a$ where $k = 1...K$, K being the number of simulated odors, and a is randomly sampled from integers from 1 to 110 for each PN and each odor.

We modeled 2000 KCs. The jth KC received N_j inputs from randomly selected PNs, where N_j was either fixed at 6 or sampled from a Gaussian distribution with mean 6 and standard deviation 1.7 (rounded to the nearest integer; minimum 2, maximum 11), based on experimental measurements (4, 5). KC claws sample PNs with replacement (5, 6), so the number of unique PNs sampled by a KC could be lower than N_j. Although more recent results show that PN-KC connectivity is not entirely random, as KCs that receive inputs from a certain group of food-odor-responsive glomeruli are slightly more likely to receive other inputs from that same group (5, 6), we judged that attempting to model this non-randomness would not add to the realism of our model given that we modeled only 24 (out of ≈50) glomeruli.

The connection from the ith PN to the jth KC had strength w_{ji}, which was 0 for non-connected neurons, and for connected neurons was either fixed at 1, sampled from a log-normal distribution ($\mu = -0.0507$ and $\sigma = 0.3527$, based on (7)), or tuned by one of the methods described below. Weights were added for duplicate connections (i.e., KCs connected more than once to the same PN). KCs received inhibition from APL (modeled as pseudo-feedforward for simplicity), with a gain that was either constant across all KCs (α) or tuned individually as described below (α_j). The KCs' spiking thresholds θ_j were either constant across all KCs, or sampled randomly from a Gaussian distribution with coefficient of variation 0.26, based on experimental measurements of the difference between spiking threshold and resting potential in 17 KCs (7). These spiking thresholds were subject to a scaling factor C_θ to achieve the correct average coding level (see below). Thus, the activity of the jth KC for the kth odor, y_j^k, was

$$y_j^k = Relu\left(\sum_{i=1}^{24} w_{ji} x_i^k - \alpha \sum_{j=1}^{M} \sum_{i=1}^{24} w_{ji} x_i^k - C_\theta \theta_j\right) \qquad [3]$$

where $M = 2000$ is the number of KCs and $Relu$ is a rectified linear unit:

$$Relu(x) = \begin{cases} 0 & x \leq 0 \\ x & x > 0 \end{cases}$$

The coding level, or fraction of KCs active for each odor, averaged across odors, was defined as:

$$CL = \frac{1}{K} \sum_{k=1}^{K} \left[\frac{1}{M} \sum_{j=1}^{M} H(y_j^k) \right] \qquad [4]$$

where K and M are the number of odors and KCs, respectively and $H(x)$ is the Heaviside function:

$$H(x) = \begin{cases} 0 & \text{if } x \leq 0 \\ 1 & \text{if } x > 0 \end{cases}$$

Experimental data suggest that coding level is around 0.1 normally, and approximately double that (0.2) when inhibition is blocked (8). To match these constraints, we minimized this error function with respect to C_θ (thus preserving the coefficient of variation of thresholds across KCs, i.e., $C_\theta \theta_j$):

$$\epsilon_{CL|\alpha=0} = \frac{1}{2} \left[CL \mid_{\alpha=0} - CL_{target|\alpha=0} \right]^2 \qquad [5]$$

where $CL_{target|\alpha=0} = 0.2$ and we minimized this error function with respect to α:

$$\epsilon_{CL} = \frac{1}{2} \left[CL - CL_{target} \right]^2 \qquad [6]$$

where $CL_{target} = 0.1$.

We tuned C_θ and α using gradient optimization, using the update equations:

$$\Delta C_\theta = -\eta \frac{d\epsilon_{CL|_{\alpha=0}}}{dC_\theta} \quad [7]$$

$$\Delta \alpha = -\eta \frac{d\epsilon_{CL}}{d\alpha} \quad [8]$$

To derive the update rule for ΔC_θ, we differentiate Eq. (5) with respect to C_θ:

$$\frac{d\epsilon_{CL|_{\alpha=0}}}{dC_\theta} = \left[CL\mid_{\alpha=0} - CL_{target|\alpha=0}\right] \frac{dCL\mid_{\alpha=0}}{dC_\theta} \quad [9]$$

To differentiate CL with respect to C_θ, we need to replace the discontinuous Heaviside function with a continuous approximation. Similar to (9) a sigmoid function approximates a Heaviside at the limit $\sigma \to 0$,

$$H(x) \approx S(x) = \frac{1}{1 + e^{-\frac{x}{\sigma}}} \quad [10]$$

Hence, assuming $\sigma = 1$, we can define the coding level as:

$$CL = \frac{1}{K} \sum_{k=1}^{K} \left[\frac{1}{M} \sum_{j=1}^{M} S(y_j^k) \right] \quad [11]$$

Given the derivative of a sigmoid is:

$$\begin{aligned} S'(x) = \frac{dS(x)}{dx} &= \frac{e^{-\frac{x}{\sigma}}}{\left[1 + e^{-\frac{x}{\sigma}}\right]^2} \\ &= S(x)(1 - S(x)) \end{aligned} \quad [12]$$

Thus,

$$\begin{aligned} \frac{dCL\mid_{\alpha=0}}{dC_\theta} &= \frac{1}{K} \sum_{k=1}^{K} \left[\frac{1}{M} \sum_{j=1}^{M} \left[S'(y_j^k\mid_{\alpha=0}) \frac{dy_j^k\mid_{\alpha=0}}{dC_\theta} \right] \right] \\ &= -\frac{1}{K} \sum_{k=1}^{K} \left[\frac{1}{M} \sum_{j=1}^{M} \left[S'(y_j^k\mid_{\alpha=0}) H(y_j^k\mid_{\alpha=0}) \theta_j \right] \right] \end{aligned} \quad [13]$$

combining Eq. (9) and Eq. (13), and plugging in Eq. (7) we can get the update equation for C_θ as

$$\Delta C_\theta = \eta \left[CL\mid_{\alpha=0} - CL_{target|\alpha=0}\right] \frac{1}{K} \sum_{k=1}^{K} \left[\frac{1}{M} \sum_{j=1}^{M} \left[S'(y_j^k\mid_{\alpha=0}) H(y_j^k\mid_{\alpha=0}) \theta_j \right] \right] \quad [14]$$

For simplicity, this can be re-written using the average operator notation $\langle\rangle$ across odors (indexed by k) and KCs (indexed by j),

$$\Delta C_\theta = \eta \left[CL\mid_{\alpha=0} - CL_{target|\alpha=0}\right] \left\langle S'(y_j^k\mid_{\alpha=0}) H(y_j^k\mid_{\alpha=0}) \theta_j \right\rangle_{j,k} \quad [15]$$

Similarly, for $\Delta\alpha$ we differentiate Eq. (6) with respect to α,

$$\frac{d\epsilon_{CL}}{d\alpha} = \left[CL - CL_{target}\right] \frac{dCL}{d\alpha} \quad [16]$$

Similarly,

$$\begin{aligned} \frac{dCL}{d\alpha} &= \frac{1}{K} \sum_{k=1}^{K} \left[\frac{1}{M} \sum_{j=1}^{M} \left[S'(y_j^k) \frac{dy_j^k}{d\alpha} \right] \right] \\ &= -\frac{1}{K} \sum_{k=1}^{K} \left[\frac{1}{M} \sum_{j=1}^{M} \left[S'(y_j^k) H(y_j^k) \sum_j \sum_i w_{ji} x_i^k \right] \right] \end{aligned} \quad [17]$$

combining Eq. (16) with Eq. (17) then putting in Eq. (8),

$$\Delta\alpha = \eta \left[CL - CL_{target}\right] \frac{1}{MK} \sum_{k=1}^{K} \sum_{j=1}^{M} \left[S'(y_j^k) H(y_j^k) \sum_j \sum_i w_{ji} x_i^k \right] \quad [18]$$

and using the $\langle\rangle$ notation:

$$\Delta\alpha = \eta\left[CL - CL_{target}\right]\left\langle S'(y_j^k)H(y_j^k)\sum_j\sum_i w_{ji}x_i^k\right\rangle_{j,k} \quad [19]$$

These update equations were used to adjust values of θ and α in any random instantiation of the fly's network to match the experimentally observed coding levels. Note that because the update equation for α is the same for all j, the same equation applies when α_j is tuned for each KC (see below). In Fig. 2E and part of Fig. 3 and S2, CL_{target} was set to values > 0.1 and α was set to 0 because for $CL_{target} > 0.5$, it is impossible for $CL_{target|\alpha=0}$ to be $2CL_{target}$.

Modelling olfactory associative learning. Learning occurred through synaptic depression at the output synapse from KCs onto MBONs according to this exponential decay rule:

$$\Delta v_j = v_j(e^{-\eta y_j^k} - 1) \quad [20]$$

where v_j is the synaptic weight between the jth KC and the MBON of the 'wrong' valence and η is the learning rate. Thus, KCs active for a punished odor weaken their synapses to the approach MBON while KCs active for the rewarded odor weaken their synapses to the avoid MBON. This can be seen as the model fly learning from 'mistakes' during its training phase (10, 11).

The behavior of the fly was determined by a softmax equation:

$$P(approach) = \frac{e^{cMBON_{approach}}}{e^{cMBON_{avoid}} + e^{cMBON_{approach}}} \quad [21]$$

where the constant c governs how probabilistic or deterministic the decision-making is. At high c, the model approaches a completely deterministic model where the fly will approach the odor 100% of the time whenever the approach MBON's activity is higher than the avoid MBON's activity; at very low c, the model approaches random chance; in between, the fly's behavior is probabilistic but biased by the imbalance between the activity of the two MBONs.

We trained the model on 15 noisy trials of the odors (no repetitions) and tested it on 15 unseen noisy trials of the same odors, and calculated the accuracy as the fraction of trials in which the model behaved correctly (i.e., avoided punished odors and approached rewarded odors).

Metrics for evaluating Kenyon cell odor representations. Angular distance between two vectors A and B (here, A and B are the centroids of each cluster of noisy trials of KC responses to two odors) was calculated using:

$$\phi = \frac{2}{\pi}\arccos\frac{A\cdot B}{\|A\|\|B\|} \quad [22]$$

Dimensionality was calculated according to the equation in (12):

$$dim(\mathbf{y}) = \frac{\left(\sum_{i=1}^m \lambda_i\right)^2}{\sum_{i=1}^m \lambda_i^2} \quad [23]$$

where λ_i are the eigenvalues of the covariance matrix of $\mathbf{y}$. Whereas Litwin-Kumar et al. calculated dimensionality analytically given inputs with defined distributions, we calculated it numerically given simulated PN inputs. Because dimensionality cannot be accurately calculated with a small number of inputs (Fig. S2A), we simulated KC activity for 50,000 input odors for dimensionality calculations.

Sparseness was calculated according to (8, 13). Using the notation of this paper, the lifetime sparseness of the jth KC for a set of K odors is:

$$S_j = \frac{1}{1-\frac{1}{K}}\left(1 - \frac{\left(\sum_{k=1}^K \frac{y_j^k}{K}\right)^2}{\sum_{k=1}^K \frac{(y_j^k)^2}{K}}\right) \quad [24]$$

If a cell is completely silent, firing to no stimuli, $y_j^k = 0$ for all k and sparseness is undefined due to division by zero.

We defined the 'valence specificity' VS of a KC as the degree to which it is more active for the set of rewarded odors (R) than punished odors (P), or vice versa:

$$VS_j = \left|\frac{\sum_{k\in R} y_j^k - \sum_{k\in P} y_j^k}{\sum_{k\in R} y_j^k + \sum_{k\in P} y_j^k}\right| \quad [25]$$

Models for compensatory variability.

Parametric tuning of excitatory input weights. We approximated the probability distribution of PN-KC synaptic weights (w) using the distribution of amplitudes of spontaneous excitatory post-synaptic potentials (mini-EPSPs) in KCs, measured by (7). This experimental distribution was approximately log-normal, as has been described for cortical synapses (14, 15), so we modeled w as following a log-normal distribution. We simulated values of w such that the overall distribution of w would follow this log-normal distribution, yet individual KCs would sample w from different log-normal distributions depending on N and θ, such that KCs with lower N or higher θ would have higher w, i.e., sampling from a log-normal distribution shifted to the right (Fig. 4A1).

The probability of PN-to-KC synaptic weights could be estimated from the probability summation rule,

$$P(w) = \int_\theta \int_N P(w \mid N, \theta) P(N) P(\theta) dN d\theta \qquad [26]$$

where $P(w \mid N, \theta)$ is the conditional probability distribution of the input synaptic weights for a KC that has N claws and spiking threshold θ, sampled from probability distributions $P(N)$ and $P(\theta)$, respectively. We approximated $P(N)$ and $P(\theta)$ as the Gaussian distributions described above (see Fig. 2), and we approximated integration over θ as summation at small intervals ($\Delta\theta = 2.5$).

We modeled the constituent conditional probability distributions $P(w \mid N, \theta)$ as also being log-normal, based on previous studies which approximate the sum of log-normal distributions as another log-normal variable by matching the first two moments of the power sum and its individual log-normal contributors (16–18). This approximation holds in our case (the Kullback-Leibler Divergence metric (KLD) converged to less than 0.001).

To get the posterior lognormal distributions $P(w \mid N, \theta)$, we minimized the distance metric Kullback-Leibler Divergence (KLD) between $P(w)$ and $\int_\theta \int_N P(w \mid N, \theta) P(N) P(\theta) dN d\theta$. To implement compensatory tuning in these conditional probabilities, such that a KC with fewer inputs (lower N) or higher spiking threshold (higher θ) would have stronger inputs (higher median w), we parameterized the medians $\tilde{\mu}$ of each conditional distribution in N and θ as:

$$\tilde{\mu} = \exp(\mu) = k\sqrt{\frac{\theta}{N}} \qquad [27]$$

Thus,

$$\mu = \ln\left(k\sqrt{\frac{\theta}{N}}\right) \qquad [28]$$

$$P(w \mid N, \theta) = \frac{1}{w\sigma\sqrt{2\pi}} \exp\left(-\frac{\left(\ln(w) - \ln\left(k\sqrt{\frac{\theta}{N}}\right)\right)^2}{2\sigma^2}\right) \qquad [29]$$

We used gradient descent optimization to find the values of σ and k in Eq. 29 that would minimize the fitting error:

$$\begin{aligned} \epsilon &= KLD[P(w), \overline{P}(w)] \\ &= \int P(w) \ln\left[\frac{\overline{P}(w)}{P(w)}\right] dw \end{aligned} \qquad [30]$$

where

$$\overline{P}(w) = \int_\theta \int_N P(w \mid N, \theta) P(N) P(\theta) dN d\theta \qquad [31]$$

First, we found the optimal σ by gradient optimisation:

$$\Delta\sigma = -\eta_1 \frac{d\epsilon}{d\sigma} \qquad [32]$$

The derivative of the fitting error with respect to σ is:

$$\frac{d\epsilon}{d\sigma} = -\int \frac{d\overline{P}(w)}{d\sigma} \frac{P(w)}{\overline{P}(w)} dw \qquad [33]$$

with,

$$\frac{d\overline{P}(w)}{d\sigma} = \int_\theta \int_N \frac{dP(w \mid N, \theta)}{d\sigma} P(N) P(\theta) dN d\theta \qquad [34]$$

where $\frac{dP(w|N,\theta)}{d\sigma}$ is:

$$\frac{dP(w \mid N, \theta)}{d\sigma} = \frac{1}{w\sigma^2\sqrt{2\pi}} \exp - \frac{\left(\ln w - \ln\left(k\sqrt{\frac{\theta}{N}}\right)\right)^2}{2\sigma^2} \left(\frac{1}{\sigma^2}\left(\ln w - \ln\left(k\sqrt{\frac{\theta}{N}}\right)\right)^2 - 1\right) \qquad [35]$$

Similarly for k,

$$\Delta k = -\eta_2 \frac{d\epsilon}{dk}$$
$$\frac{d\epsilon}{dk} = -\int \frac{d\overline{P}(w)}{dk} \frac{P(w)}{\overline{P}(w)} dw \quad [36]$$

such that,

$$\frac{d\overline{P}(w)}{dk} = \int_\theta \int_N \frac{dP(w \mid N,\theta)}{dk} P(N)P(\theta)dNd\theta \quad [37]$$

with $\frac{dP(w|N,\theta)}{dk}$ given by:

$$\frac{dP(w \mid N,\theta)}{dk} = \frac{1}{kw\sigma^3\sqrt{2\pi}} \exp - \frac{\left(\ln w - \ln\left(k\sqrt{\frac{\theta}{N}}\right)\right)^2}{2\sigma^2} \left(\ln w - \ln\left(k\sqrt{\frac{\theta}{N}}\right)\right) \quad [38]$$

Starting from arbitrary values for k and σ and using small learning rates η_1 and η_2, at each iteration, the gradient descent algorithm alternated between using σ to update k and using k to update σ. We stopped the gradient descent (i.e., the algorithm converged) at $\epsilon < 0.001$.

Tuning KC input excitatory weights to equalize KC activity. In this model, we reduce the high variance in KCs' average activity levels by tuning their input synaptic weights, such that each jth KC adjusts its input synaptic weights (w_{ji}) to make its average activity level $\overline{y}_j$ reach a certain desired level A_0. Although we ended up using a simple synaptic scaling rule in the main figures (Eq. (47)), we also explored other rules based on gradient descent and describe here the mathematical relation between them. We initially analyzed this problem using an error function:

$$\epsilon = \frac{1}{2}\left[\overline{y}_j - A_0\right]^2$$
$$\overline{y}_j = \frac{1}{K}\sum_{k=1}^{K} y_j^k \quad [39]$$

where y_j^k is the jth KC's response to the kth odor calculated as in Eq. (3) and K is the number of odors. Finding the weights to minimize the error in Eq. (39) can be found by gradient optimisation,

$$\Delta w_{ji} = -\eta \frac{d\epsilon}{dw_{ji}} \quad [40]$$

with,

$$\frac{d\epsilon}{dw_{ji}} = \left[\overline{y}_j - A_0\right] \frac{1}{K} \sum_{k=1}^{K} \frac{dy_j^k}{dw_{ji}} \quad [41]$$

Taking the derivative of y_j^k w.r.t. w_{ji} yields:

$$\frac{dy_j^k}{dw_{ji}} = H(y_j^k)(x_i^k - \alpha x_i^k) \quad [42]$$

Plugging Eq. (42) in Eq. (41) gives:

$$\frac{d\epsilon}{dw_{ji}} = \left[\overline{y}_j - A_0\right] \frac{1}{K} \sum_{k=1}^{K} H(y_j^k)(x_i^k - \alpha x_i^k)$$
$$= \left[\overline{y}_j - A_0\right] \left\langle H(y_j^k)(1-\alpha)x_i^k \right\rangle_K \quad [43]$$

Hence, w_{ji} will be updated as follows:

$$\Delta w_{ji} = -\eta \left[\overline{y}_j - A_0\right] \left\langle H(y_j^k)(1-\alpha)x_i^k \right\rangle_K \quad [44]$$

The equation above means that a KC with an average activity $\overline{y}_j$ higher (lower) than A_0 will scale down (up) its input synaptic weights, w_{ji}, proportional to both the difference ($y_j^k - A_0$) and the average input activity from the ith PN. Note that in this derivation a KC must have non-zero average activity, i.e., $H(y_j^k) = 1$ for at least one odor, for its weights to be updated. We believe such a rule would be biologically implausible, as there should not be a discontinuity between a silent KC and a nearly silent KC. To allow totally silent KCs (which have only subthreshold activity) to update their weights in the same way as active KCs, we heuristically apply the following rule:

$$\Delta w_{ji} = -\eta \left[\overline{y}_j - A_0\right] \left\langle (1 - H(y_j^k))(1-\alpha)x_i^k \right\rangle_K \quad [45]$$

Adding (44) and (45) we obtain:

$$\Delta w_{ji} = -\eta \left[\overline{y}_j - A_0\right] \left\langle (1-\alpha)x_i^k \right\rangle_K \quad [46]$$

The rule has a fixed point $\overline{y}_j = A_0$ since $\left\langle (1-\alpha)x_i^k \right\rangle_K > 0$. Note that we apply the constraint $w_{ji} \geq 0$. How updates for $w_{ji} = 0$ are treated depends on the reason why $w_{ji} = 0$: if the ith PN and jth KC are not connected, then the update is not applied. But if they were originally connected and the update rule pushed w_{ji} to zero, the update rule will continue to be applied.

To test whether performance is affected by adding the heuristic term to allow silent KCs to update their weights, we compared the performance using update rule Eq. (44) vs. Eq. (46). The rule without the heuristic performed significantly worse than the rule with the added heuristic for activating silent KCs (Fig. S3A). This means that a formally derived update rule for w was not enough, since it would not equalize activity for all KCs (silent KCs will remain silent) and would not enhance the population coding as in the heuristic rule.

We further noted that Eq. (46) contains a factor x_i^k meaning that the update to w_{ji} depends on the average input activity from the ith PN. As this rule makes the biological interpretation more complex (the synaptic update depends on both pre- and post-synaptic activity), we also tested a simplified rule where synaptic changes depend only on the average KC activity:

$$\Delta w_{ji} = -\eta \left[\overline{y}_j - A_0\right] \quad [47]$$

This simplification did not affect memory performance or the tuned distribution of weights (Fig. S3A-C), but it improved the robustness of the model to novel odor environments (Fig. S3D). This improvement in the model robustness might be because including the extra factor x_i^k in the learning rule caused the model to be overfitted to the tuning environment. Therefore, we used Eq. (47) for the results presented in the main figures, as it is simpler and produces better performance, despite not being formally derived from an error function. As with Eq. (46), this update rule has a fixed point $\overline{y}_j = A_0$.

Because KC claws sampled PNs with replacement, some KCs had 'duplicate' inputs from the same PN. For these weights, we initialised w_{ji} at double the normal level before beginning optimization. When plotting the distribution of values of w in Fig. 4D, we split these 'duplicate' weights into two weights of half the strength, on the basis that we were comparing our w values to amplitudes of spontaneous EPSPs from (7), and in a KC with two claws connected to different boutons of the same PN, spontaneous EPSPs from the two claws would likely occur at different times and thus be counted separately.

Tuning KC input inhibitory weights to equalize average KC activity. In this model, we model each KC as adjusting its individual input inhibitory synaptic weights from APL, to match its average activity level $\overline{y}_j$ to a certain desired level A_0. We minimize the error function in Eq. (39) by adjusting α_j instead of w_{ji}:

$$\Delta \alpha_j = -\eta \frac{d\epsilon}{d\alpha_j} \quad [48]$$

$$\frac{d\epsilon}{d\alpha_j} = \left[\overline{y}_j - A_0\right] \frac{1}{K} \sum_{k=1}^{K} \frac{dy_j^k}{d\alpha_j} \quad [49]$$

Differentiating y_j^k with respect to α_j yields

$$\frac{dy_j^k}{d\alpha_j} = H(y_j^k) \left[-\sum_{j=1}^{M} \sum_{i=1}^{24} w_{ji} x_i^k \right] \quad [50]$$

Plugging Eq. (50) in Eq. (49) gives,

$$\begin{aligned} \frac{d\epsilon}{d\alpha_j} &= \left[\overline{y}_j - A_0\right] \frac{1}{K} \sum_{k=1}^{K} H(y_j^k) \left[-\sum_{j=1}^{M} \sum_{i=1}^{24} w_{ji} x_i^k \right] \\ &= \left[\overline{y}_j - A_0\right] \left\langle H(y_j^k)(-\sum_{j=1}^{M} \sum_{i=1}^{24} w_{ji} x_i^k) \right\rangle_K \end{aligned} \quad [51]$$

Therefore,

$$\Delta \alpha_j = \eta \left[\overline{y}_j - A_0\right] \left\langle H(y_j^k)(\sum_{j=1}^{M} \sum_{i=1}^{24} w_{ji} x_i^k) \right\rangle_K \quad [52]$$

Similar to the previous section, we assume that weight changes for silent neurons happen in the same way as for active neurons:

$$\Delta \alpha_j = \eta \left[\overline{y}_j - A_0\right] \left\langle (1 - H(y_j^k))(\sum_{j=1}^{M} \sum_{i=1}^{24} w_{ji} x_i^k) \right\rangle_K \quad [53]$$

.

Adding (52) and (53) we obtain the inhibitory plasticity rule allowing KCs to achieve equal average activity:

$$\Delta\alpha_j = \eta\left[\overline{y}_j - A_0\right]\left\langle \sum_{j=1}^{M}\sum_{i=1}^{24} w_{ji}x_i^k \right\rangle_K \qquad [54]$$

Given that $\left\langle \sum_j \sum_i w_{ji}x_i^k \right\rangle_K$ is a constant as w_{ji} is not updated in this model, this term can be subsumed into the learning rate, so this equation reduces to:

$$\Delta\alpha_j = \eta\left[\overline{y}_j - A_0\right] \qquad [55]$$

Besides the homeostatic tuning of the APL inhibitory feedback values, these individual values of α_j also have to satisfy the sparsity constraint in Eq. (5). Therefore, the learning rule for these inhibitory weights requires simultaneously optimizing both error functions, Eq. (5) and Eq. (39). Thus combining Eq. (55) and the derivative of the sparsity constraint (CL=10%) with respect to each value of α_j,

$$\Delta\alpha_j = \eta_1\left[\overline{y}_j - A_0\right] - \eta_2\frac{d\epsilon_{CL}}{d\alpha_j} \qquad [56]$$

$$\Delta\alpha_j = \eta_1\left[\overline{y}_j - A_0\right] - \eta_2\left[CL - CL_{target}\right]\frac{dCL}{d\alpha_j} \qquad [57]$$

where

$$\frac{dCL}{d\alpha_j} = -\frac{1}{MK}\sum_{k=1}^{K}\left[S'(y_j^k)H(y_j^k)\sum_{j=1}^{M}\sum_{i=1}^{24} w_{ji}x_i^k\right] \qquad [58]$$

Combining Eq. (57) with Eq. (58),

$$\Delta\alpha_j = \eta_1\left[\overline{y}_j - A_0\right] + \eta_2\left[CL - CL_{target}\right]\left\langle S'(y_j^k)H(y_j^k)\sum_{j=1}^{M}\sum_{i=1}^{24} w_{ji}x_i^k\right\rangle_k \qquad [59]$$

We tested re-parameterizing α_j into $C_\alpha\alpha_j$ where C_α is tuned across all KCs to adjust coding level while α_j is tuned individually to equalize KC activity levels, but this had no effect on memory performance, so we kept the simpler model formulation.

Tuning KC spiking thresholds to equalize average KC activity. In this compensatory technique, we tune individual KCs' spiking thresholds θ_j to achieve equal average activity across the KC population. Starting with arbitrary initial values, each KC adjusts its spiking threshold so its average activity across K odors reaches a target level, A_0, by minimizing the error in average activity as in Eq. (39) by gradient optimization:

$$\begin{aligned}\Delta\theta_j &= -\eta\frac{d\epsilon}{d\theta_j}\\ \frac{d\epsilon}{d\theta_j} &= \left[\overline{y}_j - A_0\right]\frac{1}{K}\sum_{k=1}^{K}\frac{dy_j^k}{d\theta_j}\end{aligned} \qquad [60]$$

Differentiating y_j^k, the expression in Eq. (3), with respect to θ_j yields

$$\frac{dy_j^k}{d\theta_j} = H(y_j^k)\left[-C_\theta\right] \qquad [61]$$

Plugging Eq. (61) in Eq. (60) gives,

$$\begin{aligned}\frac{d\epsilon}{d\theta_j} &= \left[\overline{y}_j - A_0\right]\frac{1}{K}\sum_{k=1}^{K}H(y_j^k)\left[-C_\theta\right]\\ &= -\left[\overline{y}_j - A_0\right]C_\theta\left\langle H(y_j^k)\right\rangle_k\end{aligned} \qquad [62]$$

Therefore,

$$\Delta\theta_j = \eta\left[\overline{y}_j - A_0\right]C_\theta\left\langle H(y_j^k)\right\rangle_k \qquad [63]$$

Similar to Eq. (45), we assume that spiking thresholds are updated for silent KCs as well:

$$\Delta\theta_j = \eta\left[\overline{y}_j - A_0\right]C_\theta\left\langle (1 - H(y_j^k))\right\rangle_k \qquad [64]$$

Adding (63) and (64) we obtain the spiking thresholds plasticity rule allowing KCs to achieve equal average activity:

$$\Delta\theta_j = \eta C_\theta\left[\overline{y}_j - A_0\right] \qquad [65]$$

Tuning spiking thresholds to equalize KCs response probabilities. We tested an alternative strategy to tune θ suggested in (19): to equalize not $\overline{y}_j$ but rather the average response probability of each KC across K odors without inhibition, P_j, i.e.:

$$P_j = \frac{1}{K}\sum_{k=1}^{K} H(y_j^k \mid_{\alpha=0}) \qquad [66]$$

As in Eq. (5), we set this target response probability, $P_j^{target}|_{\alpha_j=0}$, to 0.2 to match experimental findings that blocking inhibition approximately doubles response probability (8). We minimized the error function:

$$\epsilon = \frac{1}{2}\left[P_j - P_j^{target}|_{\alpha_j=0}\right]^2 \qquad [67]$$

by adjusting θ_j by gradient optimization:

$$\begin{aligned}\Delta\theta_j &= -\eta\frac{d\epsilon}{d\theta_j}\\ \frac{d\epsilon}{d\theta_j} &= \left[P_j - P_j^{target}|_{\alpha_j=0}\right]\frac{dP_j}{d\theta_j}\end{aligned} \qquad [68]$$

To differentiate P_j, as in Eq. (13), we approximated the discontinuous Heaviside function with a sigmoid:

$$\begin{aligned}\frac{dP_j}{d\theta_j} &= \frac{1}{K}\sum_{k=1}^{K}\frac{dS(y_j^k \mid_{\alpha=0})}{d\theta_j}\\ \frac{dS(y_j^k \mid_{\alpha=0})}{d\theta_j} &= S'(y_j^k \mid_{\alpha=0})\frac{dy_j^k \mid_{\alpha=0}}{d\theta_j}\end{aligned} \qquad [69]$$

Recalling the formula of y_j^k in Eq. (3), it follows

$$\frac{dy_j^k \mid_{\alpha=0}}{d\theta_j} = -C_\theta H(y_j^k) \qquad [70]$$

Combining Eq. (70) with Eq. (69), and plugging in Eq. (68),

$$\frac{d\epsilon}{d\theta_j} = -\left[P_j - P_j^{target}\mid_{\alpha=0}\right] C_\theta \left\langle S'(y_j^k \mid_{\alpha=0}) H(y_j^k \mid_{\alpha=0})\right\rangle_K \qquad [71]$$

Thus, θ_j values are updated by,

$$\Delta\theta_j = \eta C_\theta \left[P_j - P_j^{target}\mid_{\alpha=0}\right] \left\langle S'(y_j^k \mid_{\alpha=0}) H(y_j^k \mid_{\alpha=0})\right\rangle_K \qquad [72]$$

As in Eq. (45), Eq. (64) and Eq. (53), we can write a symmetric rule for silent KCs:

$$\Delta\theta_j = \eta C_\theta \left[P_j - P_j^{target}{}_{|\alpha=0}\right] \left\langle S'(y_j^k \mid_{\alpha=0})(1 - H(y_j^k \mid_{\alpha=0}))\right\rangle_K \qquad [73]$$

Adding Eq. (73) and Eq. (72) leads to an activity-dependent update rule for θ_j, given all the incoming input odors:

$$\Delta\theta_j = \eta C_\theta \left[P_j - P_j^{target}{}_{|\alpha=0}\right] \left\langle S'(y_j^k \mid_{\alpha=0})\right\rangle_K \qquad [74]$$

In this model, the sparsity constraint $CL_{target|\alpha=0} = 0.2$ is satisfied by $P_j^{target}|_{\alpha_j=0} = 0.2$, because coding level equals the average of response probabilities across KCs:

$$\begin{aligned}CL &= \frac{1}{K}\sum_{k=1}^{K}\left(\frac{1}{M}\sum_{j=1}^{M} H(y_j^k)\right)\\ &= \frac{1}{M}\sum_{j=1}^{M}\left(\frac{1}{K}\sum_{k=1}^{K} H(y_j^k)\right)\\ &= \langle P_j\rangle_j.\end{aligned} \qquad [75]$$

Optimization of the multiple objective functions. As noted above, homeostatic tuning of w_{ji}, θ_j, or α_j needs to happen while maintaining the sparsity constraints, Eq. (5) and Eq. (6). (It is important to note that the homeostatic update rules are meant to represent a biological process while the sparsity constraints merely fit our model to experimental data and stand in for unknown processes that lead to a coding level of 0.1.) Since these activity-equalizing tunings both depend on and change the network's sparsity level, we used a sequential optimization approach to optimize each objective function, O_i, at a time. For each i, we find the optimal parameters $\{P_i\}$ minimizing an objective O_i, using the current estimates of the other parameters $\{P_j\}$ from all the other objectives, $\{O_j\}$ where $j \neq i$. The algorithm iterates for all i to minimise each of the objective functions, until it reaches a global minimum where the errors from all of the objective functions fall below a certain tolerance, τ_O.

Given an initial estimate for C_θ, α, θ_j and w_{ji}, the algorithm goes as follows:

Algorithm 1 Tuning of KCs parameters to equalize activity while constraining coding level

C_θ, α, parameters to be tuned for activity equalization $[w_{ji}$ or $\theta_j]$

Initialize C_θ=1, α=0, ϵ_1= ϵ_2=1, $\boldsymbol{\varepsilon_3}$= $\bar{1}$, τ_1=0.2 τ_2=0.01, $\boldsymbol{\tau_3}$= 0.06$\mathbf{A_0}$
Initialize tuned parameter for activity equalization $\{w_{ji}$ or $\theta_j\} \in$ U[0,1]
while any in $[\epsilon_1, \epsilon_2, \boldsymbol{\varepsilon_3}] > [\tau_1, \tau_2, \boldsymbol{\tau_3}]$ **do**
    1. Using the current values for θ_j and w_{ji}, update C_θ using Eq. (15)
    2. Using the value of C_θ from step (1) and current values for w_{ji}, and θ_j, update α using Eq. (19)
    3. Using C_θ and α from (1) and (2) respectively, update w_{ji} using Eq. (44) or θ_j using Eq. (65)
    4. Re-calculate the errors for the three objectives, Eq. (5), Eq. (6) and Eq. (39):
    $\{\epsilon_1 =\mid \frac{CL|_{\alpha=0}}{CL} - 2 \mid, \epsilon_2 =\mid CL - 0.1 \mid, \boldsymbol{\varepsilon_3} =\mid \mathbf{\bar{y}_j} - \mathbf{A0} \mid\}$

In our implementation we initialize the parameters to be tuned for activity equalization (w_{ji}, θ_j or α_j) from a uniform random distribution $U = [0, 1]$ (the non-tuned parameters follow the distributions in Fig. 2). In addition, we set the error for the first and second sparsity constraint, Eq. (5) and Eq. (6), to be τ_1=$\mid \frac{CL|_{\alpha=0}}{CL} - 2 \mid$=0.2, while τ_2=$\mid CL - 0.1 \mid$=0.01 respectively. This means allowing the coding level without and with the APL feedback to fall within $[1.8CL \leq CL\mid_{\alpha=0} \leq 2.2CL]$, and $[0.09 \leq CL \leq 0.11]$ respectively. For the activity equalization objective, the error $\boldsymbol{\varepsilon_3}$ is a column vector of size M, of the differences between the target average activity value A_0, and the current average activity for each KC, $\bar{y}_j$. This objective function is satisfied when all the values in the vector $\boldsymbol{\varepsilon_3}$ are less than 6% of the target activity.

Note that in the inhibition-tuning model, we tune the same parameter, α_j (a vector of M values instead of a constant), to jointly satisfy both the sparsity and the activity-equalization objectives. In this case, step (3) above is removed and step (2) updates α_j using Eq. (59).

In the model where we tune θ_j to equalize response probability rather than average activity (Fig. S4), equalizing response probability without inhibition to 0.2 also solves the coding level constraint (Eq. (75)). Thus, in this case, the algorithm iterates between 2 steps: (1) update θ_j according to Eq. (74), (2) use these values to update α according to Eq. (19), as follows,

Algorithm 2 Tuning of KCs spiking thresholds to equalize response probabilities

$C_\theta, \alpha, [\theta_j]$ to be tuned for equalizing KCs response probabilities

Initialize: $[C_\theta$=1, α=0, $\boldsymbol{\epsilon_1}$= ϵ_2=1, $\boldsymbol{\tau_1}$=0.2, τ_2=0.01$]$
Initialize $[\theta_j] \in$ U[0,1]
while any in $[\epsilon_1, \epsilon_2] > [\tau_1, \tau_2]$ **do**
    1. update θ_j using Eq. (74)
    2. Using these new values of θ_j in step (1), update α using Eq. (19)
    3. Re-calculate the errors for the two objectives, Eq. (67) and Eq. (6):
    $\{\epsilon_1 =\mid P_j - P_j^{target}|_{\alpha_j=0} \mid, \epsilon_2 =\mid CL - 0.1 \mid\}$

In our optimization pipeline, there is a potential problem in the models where KC activity is equalized by tuning α_j or θ_j. In these models w_{ji} is not tuned, so for values of A_0 that are too high relative to values of w_{ji}, excitation will be too low to reach the high targets given the constraints $C_\theta \theta_j > 0$, $CL = 0.1$ and $CL\mid_{\alpha=0}= 0.2$, meaning the algorithm does not converge. (This is not a problem when tuning w_{ji} because w_{ji} can go arbitrarily high, whereas thresholds cannot go below zero.) Therefore, w_{ji} values must be chosen in a sensible range relative to A_0 (keeping in mind that the value of A_0 is arbitrary: see below). Rather than further complicating the objective cost functions by introducing a tunable scaling factor for w_{ji}, we found that in practice the algorithm converged if w_{ji} values (starting from a log-normal distribution with $\mu = -0.0507$, $\sigma = 0.3527$) were multiplied by $\frac{A_0}{CL}$ (where $CL = 0.1$). The target activity A_0 is arbitrary because if parameters can be found to satisfy our model constraints ($\bar{y}_j = A_0$, $CL = 0.1$ and $CL\mid_{\alpha=0}= 0.2$) for a particular $A_0 > 0$, then a solution also exists for

$\overline{y}_j = cA_0$ for any $c > 0$, because:

$$
\begin{aligned}
cy_j^k &= c\,Relu\Big(\sum_{i=1}^{24} w_{ji}x_i^k - \alpha_j \sum_{j=1}^{M}\sum_{i=1}^{24} w_{ji}x_i^k - C_\theta\theta_j\Big) \\
&= Relu\Big(\sum_{i=1}^{24} (cw_{ji})x_i^k - \alpha_j \sum_{j=1}^{M}\sum_{i=1}^{24} (cw_{ji})x_i^k - cC_\theta\theta_j\Big)
\end{aligned} \qquad [76]
$$

That is, to scale $\overline{y}_j$ by a factor c, one need only scale the parameters w_{ji} and C_θ by c. In other words, only the relative magnitudes of A_0, w_{ji} and C_θ, not the absolute magnitudes, are meaningful. Thus, when comparing the distributions of w_{ji} and θ to their experimental equivalents in Fig. 4, S3 and S4, we uniformly scaled all w_{ji} and θ values to make their mean match the experimental mean, and present α values in arbitrary units.

Robustness analysis. Of the 110 odors tested in (1), we took the four chemical classes with the most odors (acids, terpenes, alcohols and esters), so that tuning parameters on a single class would provide a reasonable number of odors (at least 15). Because each class had different numbers of odors, and the memory task is more difficult when more odors need to be classified, we equalized the number of odors in each task by randomly sampling 15 odors from those classes that had more than 15 members (terpenes, 16; alcohols, 18; esters, 24), with a different random sampling for each model instantiation. Because of the small number of odors used for tuning, it was not always possible to equalize the activity of every single KC, so we allowed a maximum of 5 KCs to fall outside a ±7% bound on average activity.

Connectome analysis. KC neurite skeletons and connectivity were downloaded from the hemibrain connectome v. 1.1 (20). KCs (excluding those that receive significant non-olfactory input) were selected as neurons whose 'type' field was `KCg-m`, `KCab-c`, `KCab-m`, `KCab-s`, `KCa'b'-ap2` or `KCa'b'-m`. PN inputs for a KC were identified as neurons whose 'type' field included `adPN`, `lPN` or `vPN` (NB: some of these, e.g., vPNs, do not project to the mushroom body and so were never counted) and that formed more than 2 synapses with the KC (see Fig. 6B). KCs with truncated skeletons lacking the dendritic tree were excluded. The posterior boundary of the peduncle was the most posterior node in a skeleton annotated as being in the 'PED(R)' region of interest (annotations at https://storage.cloud.google.com/hemibrain/v1.1/hemibrain-v1.1-primary-roi-segmentation.tar.gz). The boundary between the calyx and peduncle regions in the hemibrain was defined by innervation by PNs (or lack thereof). The distance from this point to each PN-KC synapse along the KC's neurite skeleton (i.e., not the Euclidean distance) was measured as described in (21).

Code availability. Modeling and connectome analysis were carried out using custom code written in MATLAB, which is available at `https://github.com/aclinlab/CompensatoryVariability`.

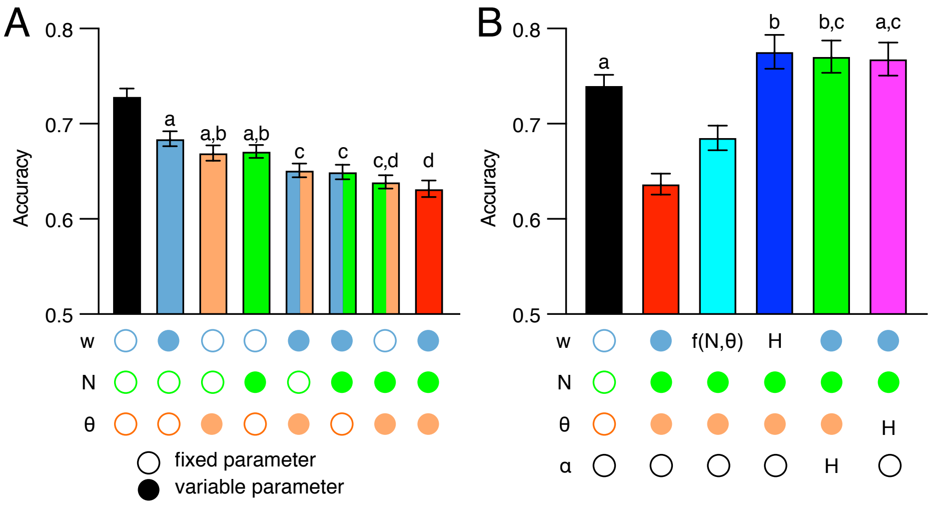

Fig. S1. Similar analyses to Fig. 2 and 4, using the original 110 odor responses from (1). **(A)** Inter-KC variability degrades the memory performance. **(B)** Compensation as in Fig. 4 improves memory performance. n = 30 (A) or 20 (B) model instances with different random PN-KC connectivity; error bars, 95% confidence interval. The indeterminacy constant c from the softmax equation was set to 10. Bars within a graph that do not share the same letter annotation are significantly different, $p < 0.05$, Mann-Whitney or Wilcoxon test as in Fig. 2, 4.

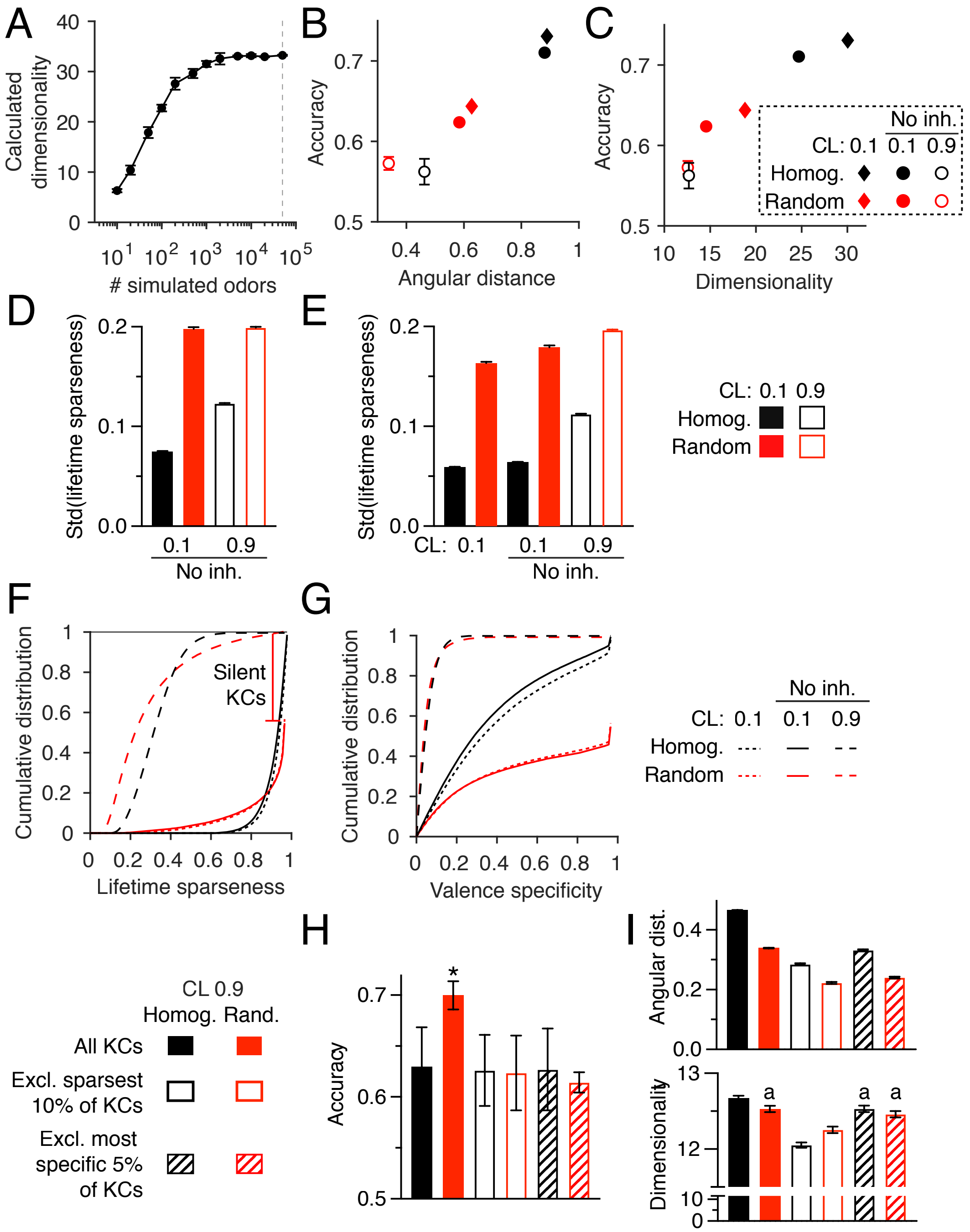

Fig. S2. Additional metrics supporting Figure 3. **(A)** Dimensionality can be estimated numerically using Eq. (23) given sufficient simulated inputs (dashed line = 50,000, the number used here). Calculations here on the homogeneous model, coding level = 0.1, with inhibition. **(B-C)** As in Fig. 3A,B, except models trained to discriminate 100 odors instead of 20 odors. The homogeneous model has higher angular distance and dimensionality than the random model ($p < 0.05$, Mann-Whitney test), matching the performance difference when coding level = 0.1, but the opposite trend to performance when coding level = 0.9. **(D-E)** The random model has greater standard deviation of lifetime sparseness across KCs, compared to the homogeneous model, in all conditions tested (coding level 0.1 or 0.9; with or without inhibition). Note: Inhibition was omitted for comparing coding level 0.1 vs. 0.9 because our model was constrained to have the coding level without inhibition be double the coding level with inhibition, which is impossible when the coding level with inhibition is 0.9. **(F-G)** As in Fig. 3C,D, except models trained to discriminate 100 odors instead of 20 odors. Cumulative distribution function (cdf) of the lifetime sparseness (C) or valence specificity (D) of KCs in the homogeneous (black) and random (red) models, across 50 model instantiations. The gap between 1.0 and the top of the cdf represents silent KCs (lifetime sparseness undefined). At coding level 0.1, the random model has many more silent KCs, non-sparse KCs, and non-specific KCs than the homogeneous model, but at coding level 0.9, the random model has more KCs with high lifetime sparseness and more KCs with high valence specificity. **(H)** Reproduced from Fig. 3F for comparison: Removing the sparsest or most valence-specific KCs removes the performance advantage of the random model under dense coding. * $p < 0.05$, Wilcoxon signed-rank test (see Table S1). **(I)** Removing the sparsest or most valence-specific KCs generally reduces angular distance and dimensionality, but not in a way that matches the effect on performance shown in (H). Conditions are significantly different (Mann-Whitney test) unless they share a letter anotation. n=50 network instantiations; error bars, 95% confidence interval (where error bars cannot be seen, they are smaller than the symbols); performance data in B,C from the 100-odor task in Fig. 2E.

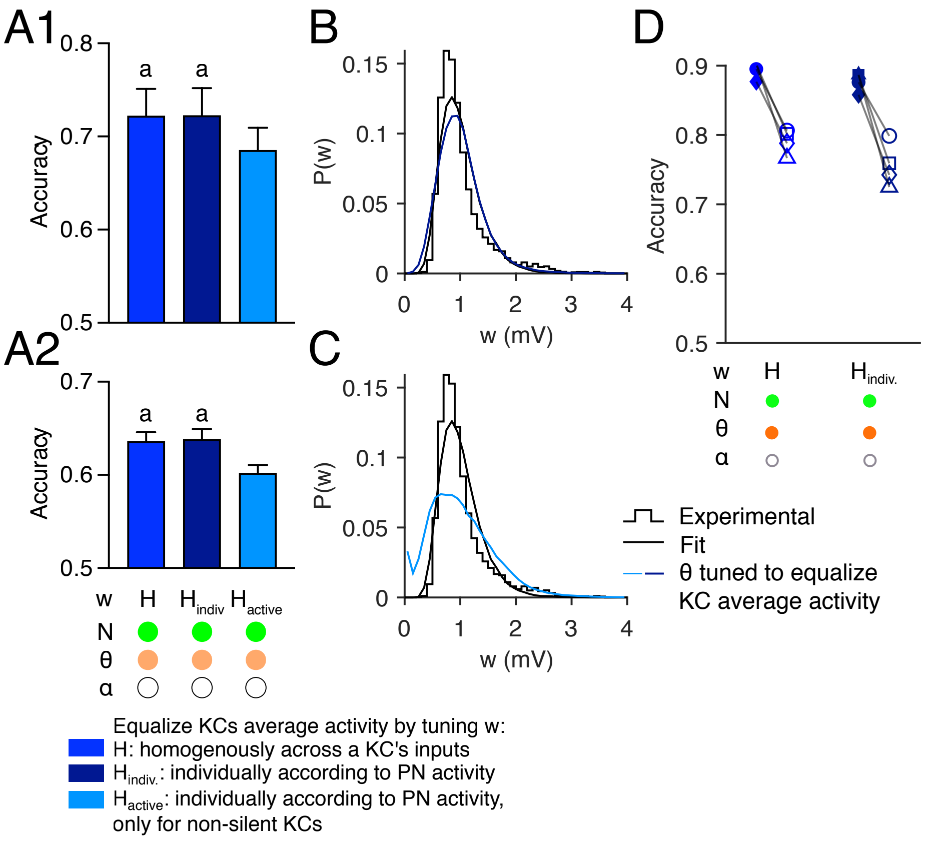

Fig. S3. Alternative update rules for tuning KCs' input excitatory weights. **(A)** Performance of different models at different indeterminacy constants (A1: $c = 10$; A2: $c = 1$). Blue, left: the method in the main figures, Eq. (47), where a given KC's input weights are all adjusted equally ('H'); dark blue, middle: Eq. (46), where a given KC's input weights are adjusted individually according to the average activity of the PN ('H_{indiv}'); light blue, right: Eq. (44), where only non-silent KCs adjust their input weights ('H_{active}'). $n = 20$ model instances with different random PN-KC connectivity. Error bars show 95% confidence interval. Bars with the same letter annotations are not significantly different from each other; all other comparisons are significant $p < 0.05$, by Wilcoxon signed-rank test with Holm-Bonferroni correction for multiple comparisons. **(B,C)** Probability distribution of the tuned excitatory weights (compare to Fig. 4E). **(D)** The 'H_{indiv}' model performs worse than the 'H' model in novel environments (see legend of Fig. 5; the drop in performance from familiar to novel environments is significantly greater for the 'H_{indiv}' model, $p < 0.05$ by Wilcoxon signed-rank test.

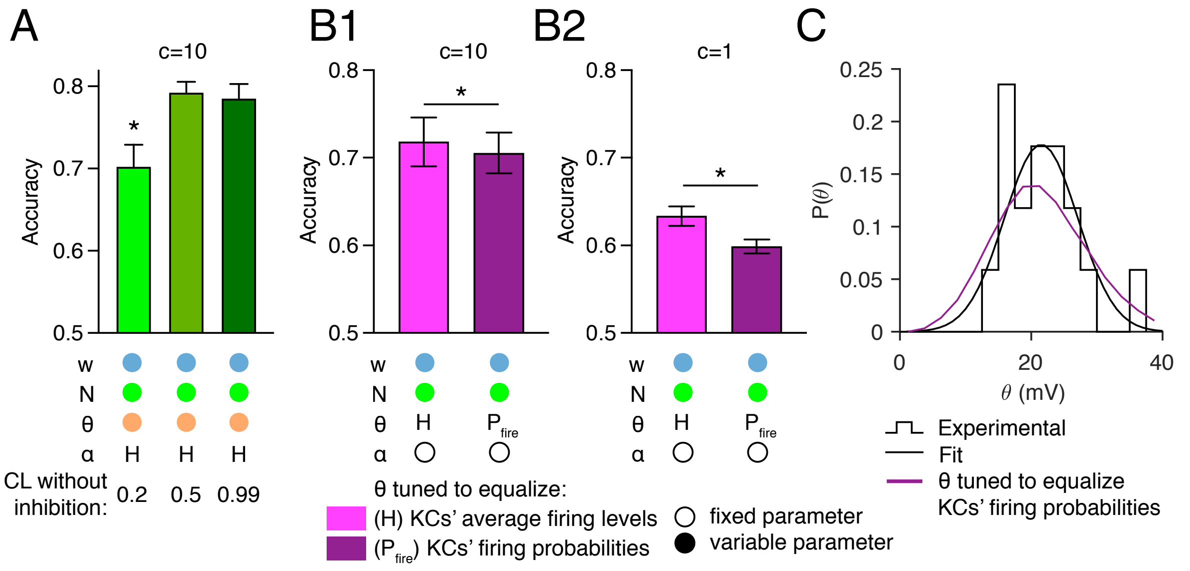

Fig. S4. Variants of activity-dependent compensation models.
(A) Tuning inhibitory weights to equalize KC average activity improves performance more when we remove the constraint that the coding level without inhibition be double (0.2) the coding level with inhibition (0.1). Coding level without inhibition was 0.2 (left, light green), 0.5 (middle, medium green) or 0.99 (right, dark green).
(B) Better performance when spiking thresholds are tuned to equalize KC average activity (magenta) rather than KC response probability (dark magenta), under both more ($c = 10$, B1) and less ($c = 1$, B2) deterministic decision-making.
(D) Probability distribution of spiking thresholds (θ) after tuning them to equalize KCs' response probabilities (compare to Fig. 4E).
$n = 20$ model instances with different random PN-KC connectivity. Error bars show 95% confidence interval. * $p < 0.05$, by Mann-Whitney test with Holm-Bonferroni correction (A) or Wilcoxon signed-rank test (B).

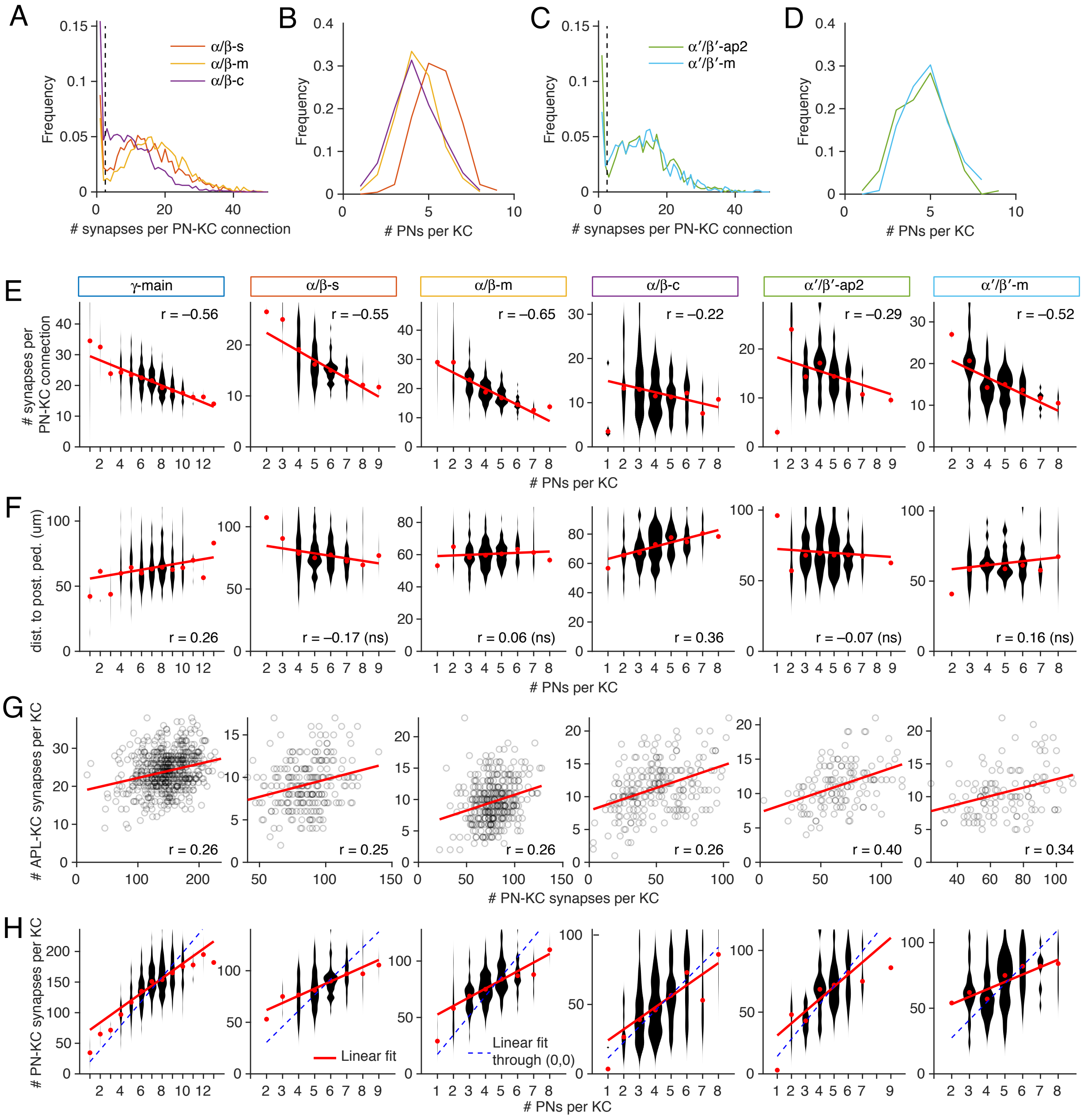

Fig. S5. Connectome analysis on all KC subtypes (γ-main, $\alpha\beta$-s, -m and -c; $\alpha'\beta'$-ap2 and -m). **(A-D)** Probability distributions of the number of synapses per PN-KC connection (A,C) and the number of input PNs per KC (B,D) in $\alpha\beta$ and $\alpha'\beta'$ KCs separated out by subtype (compare to Fig. 6E,F). **(E)** Mean number of input synapses per PN-KC connection is inversely related to the number of input PNs per KC. **(F)** Mean distance of PN-KC synapses to the posterior boundary of the peduncle (presumed spike initiation zone) is directly related to the number of input PNs per KC in γ and $\alpha\beta$-c KCs. **(G)** The number of APL-KC synapses per KC is directly related to the total number of PN-KC synapses per KC. **(H)** The number of PN-KC synapses per KCs grows sublinearly with the number of PN inputs per KC. Red dots: medians. Red lines: linear fits. Blue dashed lines: linear fits through the origin (if every PN-KC connection had the same number of synapses). Note that the red dots follow a concave function relative to both linear fits.

References

1. EA Hallem, JR Carlson, Coding of odors by a receptor repertoire. *Cell* **125**, 143–160 (2006).
2. SR Olsen, V Bhandawat, RI Wilson, Divisive normalization in olfactory population codes. *Neuron* **66**, 287–299 (2010).
3. V Bhandawat, SR Olsen, NW Gouwens, ML Schlief, RI Wilson, Sensory processing in the drosophila antennal lobe increases the reliability and separability of ensemble odor representations. *Nat. Neurosci.* **10**, 1474–1482 (2007).
4. S Caron, V Ruta, L Abbott, R Axel, Random convergence of olfactory inputs in the drosophila mushroom body. *Nature* **497**, 113–117 (2013).
5. F Li, et al., The connectome of the adult Drosophila mushroom body provides insights into function. *eLife* **9** (2020).
6. Z Zheng, et al., Structured sampling of olfactory input by the fly mushroom body. *bioRxiv*, 2020.04.17.047167 (2020).
7. GC Turner, M Bazhenov, G Laurent, Olfactory representations by drosophila mushroom body neurons. *J. Neurophysiol.* **99**, 734–746 (2008).
8. AC Lin, A Bygrave, A de Calignon, T Lee, G Miesenböck, Sparse, decorrelated odor coding in the mushroom body enhances learned odor discrimination. *Nat. Neurosci.* **17**, 559–568 (2014).
9. X Han, PE Kloeden, Sigmoidal approximations of heaviside functions in neural lattice models. *J. Differ. Equations* **268**, 5283–5300 (2020).
10. DR Chialvo, P Bak, Learning from mistakes. *Neuroscience* **90**, 1137–48 (1999).
11. JS Albus, A theory of cerebellar function. *Math. Biosci.* **10**, 25–61 (1971).
12. A Litwin-Kumar, KD Harris, R Axel, H Sompolinsky, L Abbott, Optimal degrees of synaptic connectivity. *Neuron* **93**, 1153–1164 (2017).
13. B Willmore, DJ Tolhurst, Characterizing the sparseness of neural codes. *Network: Comput. Neural Syst.* **12**, 255–270 (2001).
14. S Song, PJ Sjöström, M Reigl, S Nelson, DB Chklovskii, Highly nonrandom features of synaptic connectivity in local cortical circuits. *PLoS biology* **3**, e68 (2005).
15. G Buzsáki, K Mizuseki, The log-dynamic brain: how skewed distributions affect network operations. *Nat. Rev. Neurosci.* **15**, 264–278 (2014).
16. L Fenton, The sum of log-normal probability distributions in scattered transmission systems. *IRE Trans.Commun.Systems* **8**, 57–67 (1960).
17. S Schwartz, Y Yeh, The distribution function and moments of power sums with log-normal components. *Bell Syst. Tech. J.* **61**, 1441–1462 (1982).
18. D Dufresne, Sums of lognormals in *Actuarial Research Conference.* pp. 1–6 (2008).
19. A Kennedy, Learning with naturalistic odor representations in a dynamic model of the drosophila olfactory system. *bioRxiv* (2019).
20. LK Scheffer, et al., A Connectome and Analysis of the Adult Drosophila Central Brain. *bioRxiv* **12**, 2020.04.07.030213 (2020).
21. H Amin, AA Apostolopoulou, R Suárez-Grimalt, E Vrontou, AC Lin, Localized inhibition in the Drosophila mushroom body. *eLife* **9**, e56954 (2020).
